# A green metal-free “one-pot” microwave assisted synthesis of 1,4-dihydrochromene triazoles†

**DOI:** 10.1039/d1ra01169c

**Published:** 2021-03-09

**Authors:** Tânia M. F. Alves, Guilherme A. M. Jardim, Marco A. B. Ferreira

**Affiliations:** Centre for Excellence for Research in Sustainable Chemistry (CERSusChem), Department of Chemistry, Federal University of São Carlos – UFSCar Rodovia Washington Luís, Km 235, SP-310, São Carlos São Paulo 13565-905 Brazil marco.ferreira@ufscar.br

## Abstract

The synthesis of several 4-aryl-1,4-dihydrochromene-triazoles was achieved *via* a metal-free “one-pot” procedure using PEG400 as the sole solvent in an eco-friendly process. Using microwave irradiation, the triazole derivatives were obtained in good yields and short reaction times starting from readily accessible building blocks.

## Abstract 2

A straightforward metal-free “one-pot” microwave procedure using PEG400 as the sole solvent in an eco-friendly process for the synthesis of 4-aryl-1,4-dihydrochromene-triazoles is described.

## Graphical abstract

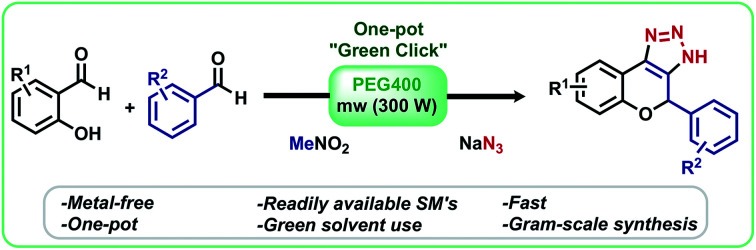

Novel green technologies play a pivotal role in modern society economics and way of life, and are being considered an important engine of transformation by directly affecting people's daily lives.^1^ In this sense, the “Click Chemistry” philosophy encompasses several features within the “Twelve Principles of Green Chemistry”, including mild reaction conditions, simple operation, simple product isolation procedures, solvent-free conditions, readily available starting materials and reagents, high yields and selectivity.^2^

Among the “Click” reaction arsenal, the formation of 1,2,3-triazoles emerged as a reliable approach for the synthesis of novel compounds, being present in several fields of science, particularly in biomedical chemistry and drug design.^3^ 1,2,3-Triazoles are useful building blocks in organic chemistry owing to their stablily to moisture, oxidation, and metabolism in organisms.^4^ Moreover, these moieties are powerful pharmacophores, playing an important role in bio-media.^5^ Mainly represented by the Cu(i) catalyzed Huisgen cycloaddition over the last years, the majority of reports englobes the use of sodium/organic azides and alkynes.^6^

Green and sustainable triazole synthesis approaches have gained the attention of chemists more recently, with highlights to metal-free conditions,^7^ heterogeneous reactions employing an immobilized catalyst^8^ and microwave irradiation.^9^ Regarding green protocols that use nitroolefins as dipolarophiles,^10^ the metal-free acid-catalyzed approach by Guan *et al.*^11^ as well as the use of palladium- and copper-supported mesoporous polyaromatic hydrocarbons by Banerjee *et al.*^12^ represent important advances in this field. However, due the lack of solubility of azides, DMSO and DMF are the solvents of choice for those transformations.

The use of water as a benign solvent in organic reactions is always appreciated as it is one of the most abundant, cheapest, and greener solvents.^13^ As another important green alternative, polyethylene glycol 400 (PEG400) has been used to enhance the solubility of organic compounds. An important feature of PEG400 is its ability to act as a crown ether,^14^ enhancing the solubility of metal-based salts such as sodium azide (Scheme 1A).

**Scheme 1:**
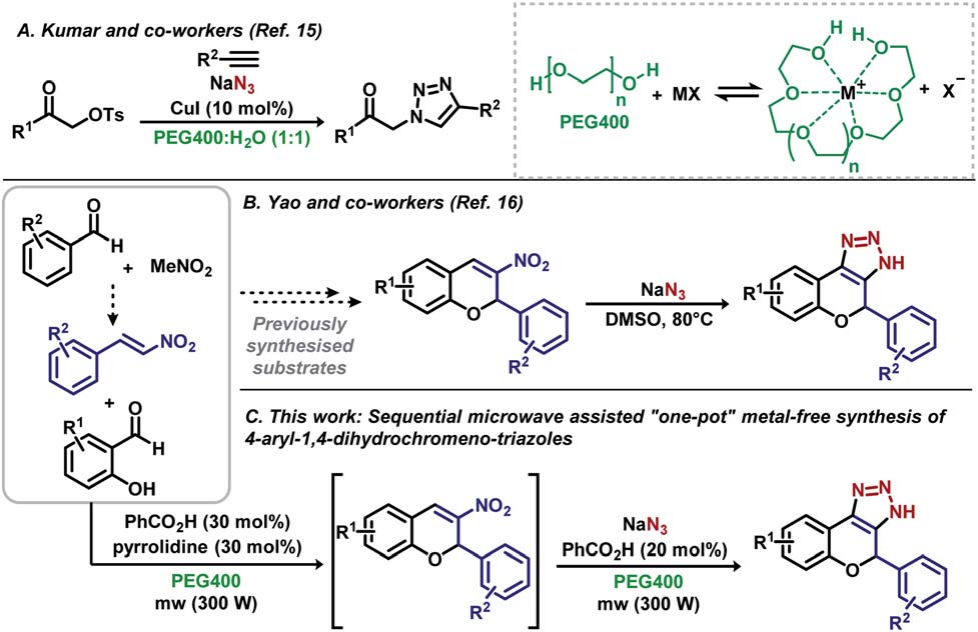
Previously reported procedures and novel microwave assisted “one-pot” metal-free reaction.

Kumar and Varma demonstrated that aqueous PEG400 is an excellent solvent for Cu(i)-catalysed Huisgen cycloadditions (Scheme 1A).^15^ In the same year, Yao *et al.* reported the synthesis of 4-aryl-1,4-dihydrochromene-triazoles from 3-nitrochromens in DMSO (Scheme 1B).^16^ In recent years, nitroolefins have been efficiently explored, allowing the direct obtention of 1*H*-1,2,3-triazoles using sodium azides with concomitant aromatization,^10–14,16^ and HNO_2_ release, which assists in the decomposition of residual NaN_3_.^10*f*^ Aiming for a more sustainable process, based on microwave irradiation and sequential metal-free “one-pot” reactions, we developed a methodology starting from simple building blocks such as substituted benzaldehydes and nitromethane, avoiding the manipulation of potentially toxic nitroalkenes, and using PEG400 as the sole solvent for the synthesis of aryl-1,4-dihydrochromene 1*H*-1,2,3-triazoles (Scheme 1C). We believe that this novel procedure can open new possibilities for the synthesis of such an important scaffold in medicinal chemistry.

Our study began with the optimization of a reaction model consisting of 1a, sodium azide and PEG400 as the solvent under microwave irradiation conditions (300 W) (Table 1-entry 1). With a reaction time of 10 min at 120 °C, a complex mixture of products was obtained. In the second attempt, temperature was maintained at 80 °C for 20 min, and 2a was obtained in 37% yield (entry 2). Based on previous reports,^11^ catalytic amounts of acids were employed, aiming at reducing the formation of 1,3,5-triphenylbenzene. The addition of *p*-toluene sulfonic acid (PTSA) resulted in a slightest yield improvement, while the use of camphor sulfonic acid (CSA) led to a decrease in yield (entries 3–4). Although an increase in the yield was not observed by increasing the concentration of the reaction medium (entry 5), a better result was observed when catalytic amounts of several acids were used under the same conditions (entry 6–9), and the best result was found for benzoic acid (62%, entry 9), with a minor increase in the reaction time. Previous results showed similar performance for acetic acid as the solvent.^10*f*^ Next, an increase in the amount of benzoic acid led to a decrease in the yield (entries 10–11) and attempts of using mixtures of solvents resulted in the reaction inhibition (entries 12–13). Furthermore, doubling the reaction concentration, decreasing the reaction temperature and raising the reaction time led to unsatisfactory results (entries 14–15).

**Table 1:** Selected optimization results

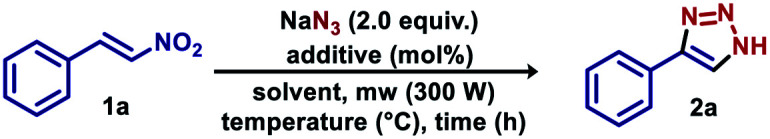						
Entry	Solvent	Temp.	Time	1a (M)	Additive (mol%)	Yield^a^ (%)
1	PEG400	120	10	0.2	—	Mixture
2	PEG400	80	20	0.2	—	37%
3	PEG400	80	20	0.2	PTSA (10)	40%
4	PEG400	80	20	0.2	CSA (10)	30%
5	PEG400	80	20	0.4	—	35%
6	PEG400	80	20	0.4	PTSA (10)	48%
7	PEG400	80	20	0.4	CSA (10)	54%
8	PEG400	80	20	0.4	TFA (10)	30%
**9**	**PEG400**	**80**	**25**	**0.4**	**PhCO**_**2**_ **H (10)**	**62%**
10	PEG400	80	25	0.4	PhCO_2_H (20)	58%
11	PEG400	80	25	0.4	PhCO_2_H (30)	48%
12	PEG400/EtOH (8 : 2)	80	35	0.4	PhCO_2_H (10)	Trace
13	PEG400/H_2_O (8 : 2)	80	35	0.4	PhCO_2_H (10)	Trace
14	PEG400	80	20	0.8	PhCO_2_H (10)	34%
15	PEG400	60	35	0.4	PhCO_2_H (10)	Trace

a All yields were isolated.

To demonstrate the effectiveness of the optimized reaction, model substrates 1b–g were previously synthesized and applied as starting materials, and resulted in derivatives 2b–g in yields lower than obtained by that the model substrate 1a (Scheme 2). Apparently, no differences were observed for *para*-substituted benzo nitroolefins with electron-donating and electron-withdrawing groups (derivatives 2b–d). The reaction proceeded smoothly for substrates bearing a methyl group in the olefin terminal carbon (derivatives 2e–g), with unexpected lower yields.

**Scheme 2:**
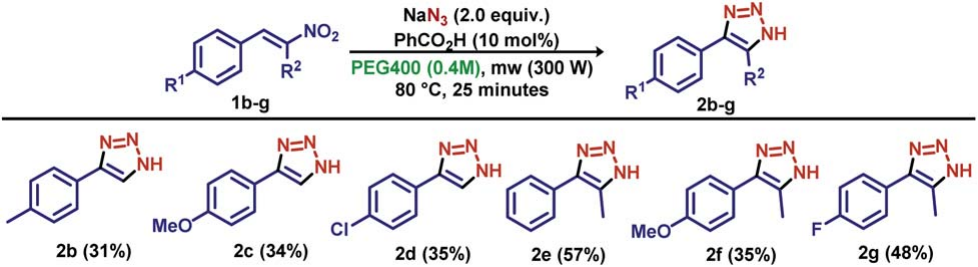
Initial results for reaction scope.

Inspired by the previous results, new experiments were planned to expand the methodology for the obtention of dihydrochromene-triazole moieties in a “one-pot” microwave procedure, which initially explored the individual elementary steps. For this endeavour, the synthesis of nitrochromene 3a was conducted using parameters obtained in the optimization steps (Scheme 3A). The reaction of 1a with salicylaldehyde at 80 °C in 35 min using acid Al_2_O_3_ gave 3a in 56% yield (entry 1). Best results were found using benzoic acid/pyrrolidine (pyrro.) (81%, entry 2) and PTSA as additives (83%, entry 3). As shown in entry 5, reaction carried out under conventional heating over 2.5 h resulted in minor yield improvements. Next, 3a was submitted to the reaction with sodium azide using different amounts of benzoic acid and PTSA to find the optimized quantity of the additives (Scheme 3B). For our delight, the reaction with 50 mol% of both acids afforded 4a in 80% and 78%, respectively after fine tuning the reaction temperature and time (10 min at 110 °C) (entries 8–9). Similar yields were obtained by performing the reaction under conventional heating over 2.5 h (entry 12). Starting from 1a, the “one pot” synthesis of 4a was accomplished in 80% yield over two steps, as exemplified in Scheme 3C.

**Scheme 3:**
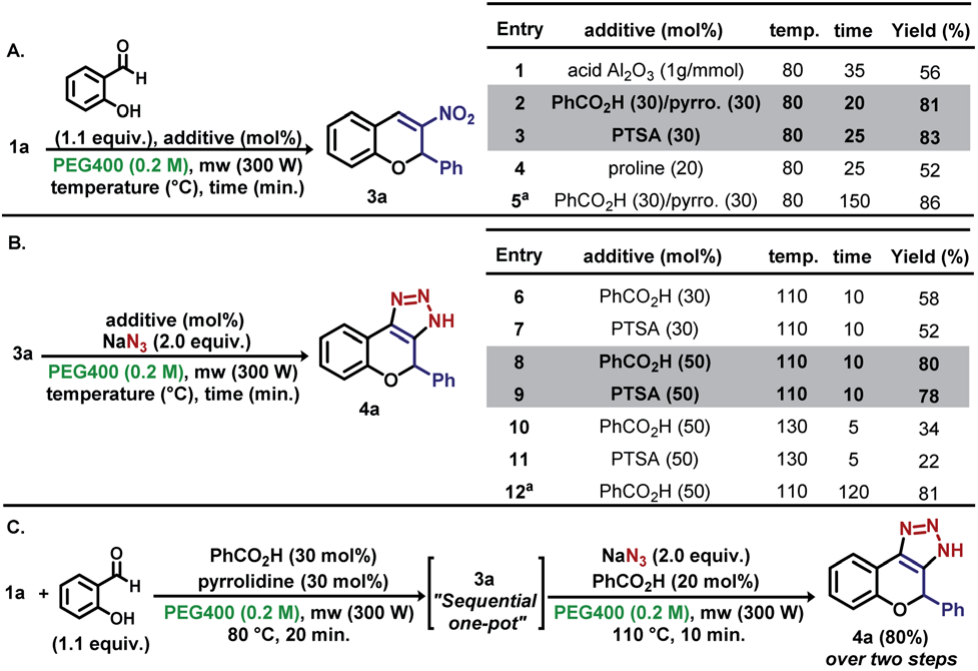
Optimization and fine tuning for the “one-pot” synthesis of 4a. ^a^Reaction conducted using conventional heating (oil bath).

Next, the sequential procedure was used to synthesize a series of novel derivatives bearing different structural features (Scheme 4). Starting from phenyl-substituted nitroolefins 1a–k, the novel sequential “one-pot” reaction was executed, which afforded derivatives 4a–k in average to high yields in a two-step procedure. The reaction proceeded smoothly with electron-withdrawing groups at the *para* position, as showed for derivatives 4d and 4g, as well as derivative 4j, which bears a nitro group at the *meta* position. A slight decrease in the yield was observed for derivatives bearing electron-donating groups at the *para* position (4b–c, 4e–f), *meta* and *ortho* positions (4i and 4k), and same behaviour was observed for derivative 4h, which bears a methoxy group at both *meta* and *para* positions. Different salicylaldehyde derivatives were also employed, which afforded derivatives 4l–o in good yields. Slight reduction in the yield was observed for both electron-withdrawing and electron-donating groups, with major decrease for brominated derivative 4n (55%) and yields ranging from 55% to 64% (Scheme 4).

**Scheme 4:**
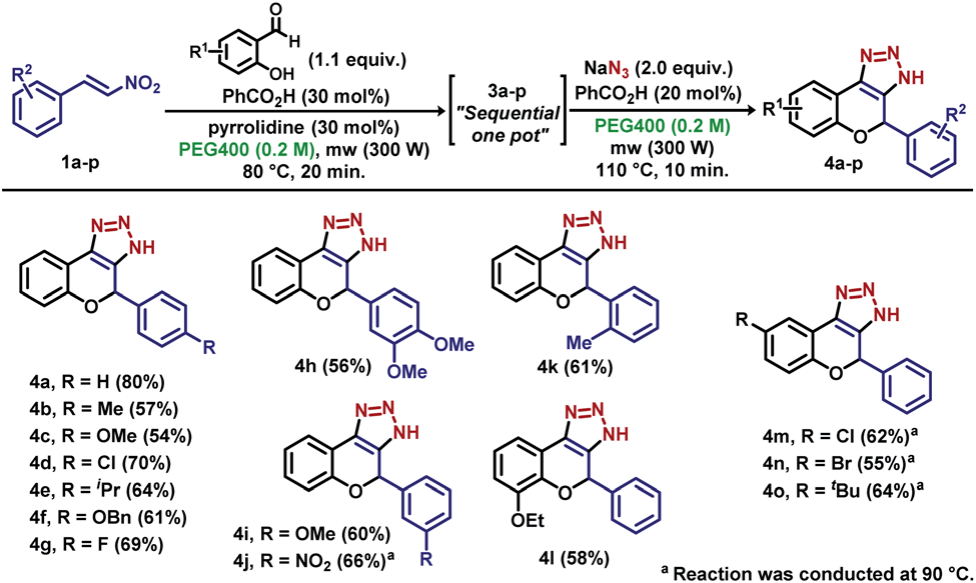
Scope for sequential “one-pot” procedure.

To expand the functionality of the methodology, the synthesis of substituted nitroolefins was conducted *via* microwave irradiation to verify the orthogonality of the reactants needed in the next steps. To our delight, substituted 2-nitrovinyl benzenes were detected *via* the TLC analysis, and further steps were carried out for the synthesis of aryl-1,4-dihydrochromeno-triazoles (Scheme 5A). Derivatives 4a, 4d and 4i were obtained in good yields per reaction step, showing that a three-step “one-pot” procedure is feasible for the synthesis of these triazole moieties.

**Scheme 5:**
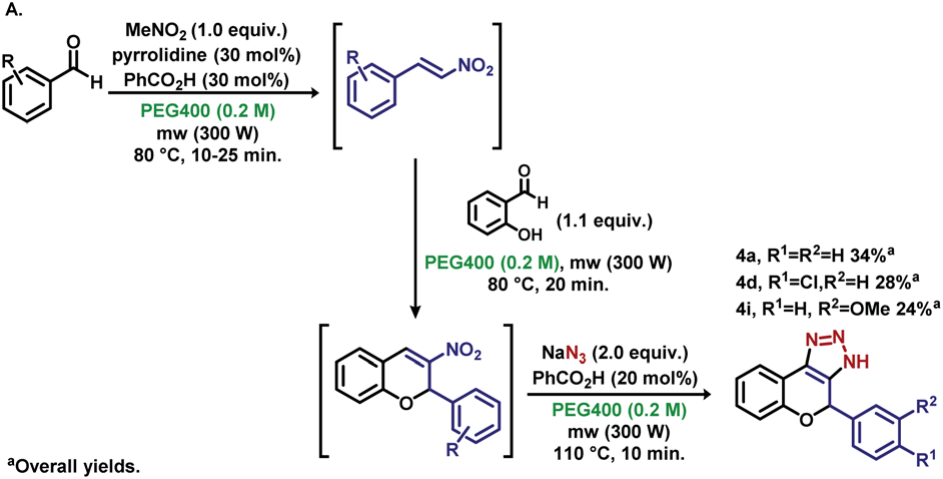
Three step “one-pot” microwave assisted synthesis of 4a, 4d and 4i.

The methodology was scaled to provide information about the robustness of microwave irradiation. As shown in Scheme 6A, scaling up to 0.2, 0.5 and 1 mmol (2.5× and 5× folder) provided 4a with almost no variation in the yield for the two-step process, although a decrease in the yield was observed for the three-step reaction. Scaling up to 2 mmol (10× folder) in the two-step reaction resulted in almost 20% less yield, and the decrease in yield was observed for larger amounts (3 mmol–15× folder, 5 mmol–25× folder and 10 mmol–50× folder). For the three-step procedure, no product was observed after scaling up to 3 mmol.

**Scheme 6:**
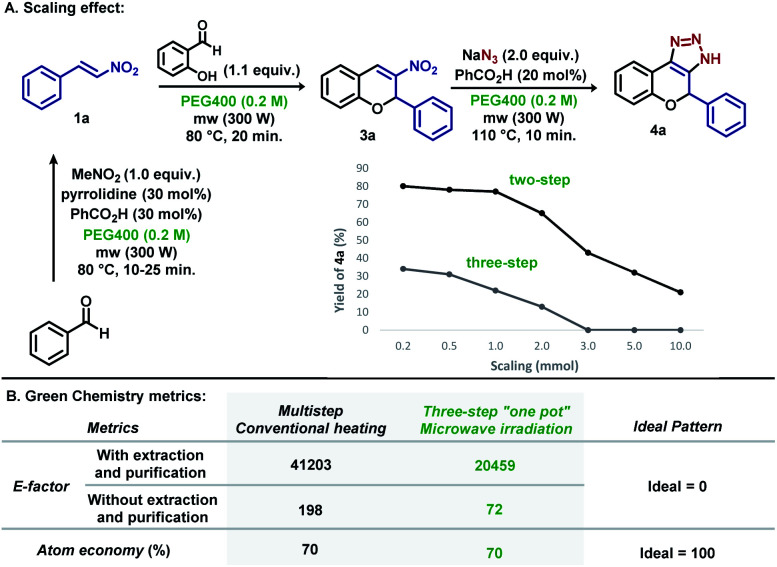
Scaling effect and green chemistry metrics for 4a.

Next, two green chemistry metrics (*E*-factor and atom economy) were calculated for the conventional multistep procedure, and the three-step microwave irradiation method (see ESI†).^17^ As shown in Scheme 6B, *E*-factor was determined for the two procedures by comparing parameters regarding the extraction and purification in both cases. It is notable that the three-step microwave irradiation procedure shows the *E*-factor value closer to the ideality mostly because it “skips” two classical extraction and purification steps. When calculation was performed in the absence of these two parameters, the method presents an *E*-factor = 72, which is mostly due to the decrease in the amount of the solvent used in the reaction steps. In both cases, the atom economy is the same, since this green chemistry metric does not consider the other reagents, solvents and catalysts used in the purification and extraction steps.

## Conclusions

In summary, a three step “one-pot” procedure was successfully developed for the synthesis of important biological motifs using PEG400 as the sole solvent in the process. All reaction steps were carefully investigated to determine the best reaction parameters encompassing the “one-pot” methodology. Considered an eco-friendly solvent, the use of PEG400, allied with microwave irradiation, provided a fast, efficient, and green reaction for obtaining dihydrochromene-triazole hybrids in good overall yields. Scaling experiments were conducted, showing the limit in which the reaction maintains its robustness. A quantitative comparison based on Green Chemistry metrics between the multistep conventional heating and “one-pot” microwave irradiation procedure showed that the second method presents advantages in terms of sustainability.

## Conflicts of interest

There are no conflicts to declare.

## Supplementary Material

RA-011-D1RA01169C-s001
