# Supplementary material for: A green metal-free “one-pot” microwave assisted synthesis of 1,4-dihydrochromene triazoles
Source: RSC Adv. 2021 Mar 9;11(17):10336–9. doi: 10.1039/d1ra01169c (PMC8695595; doi:10.1039/d1ra01169c)
Supplement: RA-011-D1RA01169C-s001 [file RA-011-D1RA01169C-s001.pdf]

Electronic Supplementary Material (ESI) for RSC Advances.
This journal is © The Royal Society of Chemistry 2021

Supporting Information

Green metal-free "one-pot" microwave assisted synthesis of 1,4-dihydrochromene triazoles

Tânia M. F. Alves [a]; Guilherme A. M. Jardim[a]; Marco A. B. Ferreira[a*]

[a]Centre for Excellence for Research in Sustainable Chemistry (CERSusChem), Department of Chemistry, Federal University of São Carlos – UFSCar, Rodovia Washington Luís, km 235, SP-310, São Carlos, São Paulo, Brazil, 13565-905.

*marco.ferreira@ufscar.br

Generalities

Reagents, when not synthesized, as well as solvents were obtained commercially and when necessary were treated according to the literature. Thin layer chromatography analysis (TLC) using fluorescent-treated silica gel 60 (F_{254}) coated aluminum plates and revealed under UV light and/or vanillin were performed to follow up the reactions. Column chromatography using silica gel 60 (230-240 mesh) was the technique of choice for purifications. ^{1}H and ^{13}C NMR spectra were recorded using the Bruker Advance 400 brand spectrometer at 400 MHz and 100 MHz, respectively, employing $CDCl_3$ or DMSO-d_6 as solvent, using tetramethylsilane (TMS) for the ^{1}H NMR spectra as a reference and for the ^{13}C spectra the solvent signal was used, with the chemical displacements (δ) reported in ppm and the coupling constants (*J*) in Hertz (Hz). The following abbreviations were used to note signal multiplicities: s - singlet; d - doublet; t - triplet; q - quartet; dd - doublet of doublets, ddt - doublet of doublet of triplets; dq - doublet of quartets; sept - septet; m - multiplet. Melting point analysis were performed on a Melting Point M-560 (BUCHI®) apparatus and high-resolution mass spectra were recorded using a Bruker model IMPACT HD™ spectrometer operating in positive mode and electrospray ionization source and quadrupole time of flight analyzer (ESI-QqTOF). Microwave assisted reactions were performed in a CEM-Discover Collmate apparatus with air flush to control flask temperature.

Experimental procedures

Preparation of nitroolefins 1a-o[1]

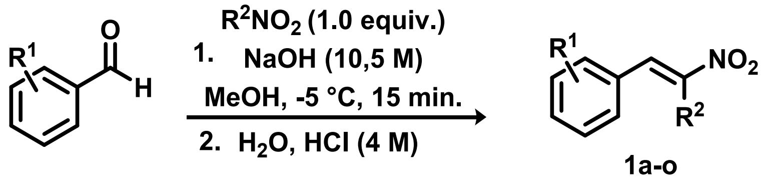

Method A: a 100 mL round bottom flask was charged with the corresponding aldehydes (10.0 mmol), nitro compounds (10.0 mmol) and methanol (4.0 mL) and the mixture was cooled to -5 °C. Then, 2.0 mL of a solution of NaOH (10.5 M) was added dropwise to the mixture, and temperature was kept at -5 °C. The mixture was vigorously stirred for 15 minutes and water (7.0 mL) was added. The resulting mixture was slowly poured in a 50

mL erlenmeyer containing a 4 M solution of HCl (6.0 mL). The yellow precipitate was collected by simple filtration and recrystallized in EtOH (6.0 mL).

Method B: A 5.0 mL resealable microwave reaction vessel was charged with benzaldehyde (20.3 μL, 0.20 mmol), nitromethane (11 μL, 1.0 equiv.), benzoic acid (7.2 mg, 30 mol%), pyrrolidine (5.5 μL, 30 mol%) and PEG400 (1.0 mL). The mixture was irradiated at 300 W with a constant temperature of 80 °C for 10 minutes. Next, water (5.0 mL) was added, and the precipitate was filtered and recrystallized in ethanol (6.0 mL).

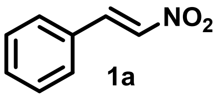

1a

(*E*)-(2-nitrovinyl)benzene (1a)[2]: yellow solid, 74% yield. **^{1}H NMR** (400 MHz, $CDCl_3$) δ: 8.00 (d, *J* = 13.7 Hz, 1H); 7.59 (d, *J* = 13.7 Hz, 1H); 7.56 – 7.42 (m, 5H).

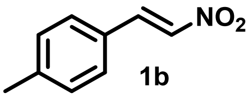

1b

(*E*)-1-methyl-4-(2-nitrovinyl)benzene (1b)[2]: yellow solid, 75% yield. **^{1}H NMR** (400 MHz, $CDCl_3$) δ: 7.98 (d, *J* = 13.6 Hz, 1H); 7.57 (d, *J* = 13.6 Hz, 1H); 7.44 (d, *J* = 8.2 Hz, 2H); 7.28-7.24 (m, 2H); 2.41 (s, 3H).

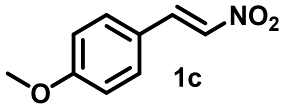

1c

(*E*)-1-methoxy-4-(2-nitrovinyl)benzene (1c)[2]: yellow solid. 66% yield. **^{1}H NMR** (400 MHz, $CDCl_3$) δ: 7.98 (d, *J* = 13.6 Hz, 1H); 7.53 (d, *J* = 13.6 Hz, 1H), 7.48 – 7.44 (m, 2H); 7.00 – 6.92 (m, 2H); 3.87 (s, 3H).

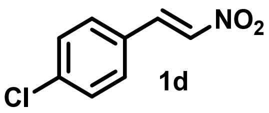

1d

(*E*)-1-chloro-4-(2-nitrovinyl)benzene (1d)[2]: yellow solid, 72% yield. **^{1}H NMR** (400 MHz, $CDCl_3$) δ: 7.96 (d, *J* = 13.7 Hz, 1H); 7.56 (d, *J* = 13.7 Hz, 1H); 7.52 – 7.41 (m, 4H).

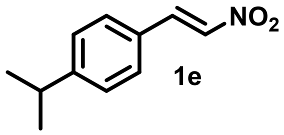

1e

(E)-1-isopropyl-4-(2-nitrovinyl)benzene (1e)[2]: yellow solid, 65% yield. **^{1}H NMR** (400 MHz, $CDCl_3$) δ: 7.99 (d, J = 13.7 Hz, 1H); 7.57 (d, J = 13.6 Hz, 1H); 7.61-7.53 (m, 2H); 7.34-7.29 (m, 2H); 3.01-2.92 (m, 1H); 1.27 (d, J = 6.9 Hz, 6H).

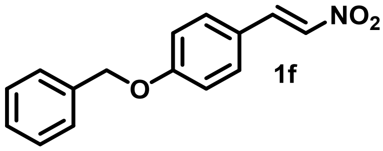

1f

(E)-1-(benzyloxy)-4-(2-nitrovinyl)benzene (1f)[2]: yellow solid, 78% yield. **^{1}H NMR** (400 MHz, $CDCl_3$) δ: 7.97 (d, J = 13.6 Hz, 1H); 7.52 (d, J = 13.6 Hz, 1H); 7.50 – 7.47 (m, 2H); 7.45 – 7.33 (m, 5H); 7.07 – 6.99 (m, 2H); 5.13 (s, 2H).

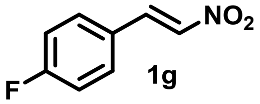

1g

(E)-1-fluoro-4-(2-nitrovinyl)benzene (1g)[2]: yellow solid, 73% yield. **^{1}H NMR** (400 MHz, $CDCl_3$) δ: 7.98 (d, J = 13.7 Hz, 1H); 7.57 (d, J = 13.7 Hz, 1H); 7.54 – 7.50 (m, 2H); 7.19 – 7.12 (m, 2H).

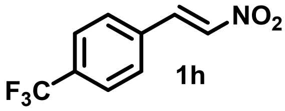

1h

(E)-1-(2-nitrovinyl)-4-(trifluoromethyl)benzene (1h)[2]: yellow solid, 68% yield. **^{1}H NMR** (400 MHz, $CDCl_3$) δ: 8.02 (d, J = 13.8 Hz, 1H); 7.74 – 7.65 (m, 4H); 7.62 (d, J = 13.7 Hz, 1H).

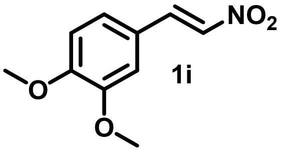

1i

(E)-1,2-dimethoxy-4-(2-nitrovinyl)benzene (1i)[2]: yellow solid, 79% yield. **^{1}H NMR** (400 MHz, DMSO-d_6) δ: 8.20 (d, J = 13.5 Hz, 1H); 8.05 (d, J = 13.5 Hz, 1H); 7.50 – 7.46 (m, 1H); 7.44 – 7.38 (m, 1H); 7.04 (d, J = 8.4 Hz, 1H); 3.81 (d, J = 5.4 Hz, 6H).

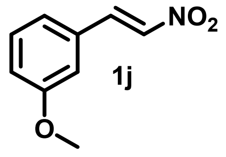

1j

(*E*)-1-methoxy-3-(2-nitrovinyl)benzene (1j)[2]**:** yellow solid, 82% yield. **^{1}H NMR** (400 MHz, $CDCl_3$) δ: 7.97 (d, *J* = 13.7 Hz, 1H); 7.57 (d, *J* = 13.7 Hz, 1H); 7.40 – 7.33 (m, 1H); 7.17 – 7.11 (m, 1H); 7.06 – 7.01 (m, 2H); 3.85 (s, 3H).

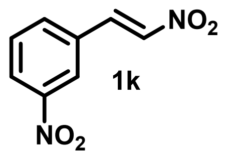

1k

(*E*)-1-nitro-3-(2-nitrovinyl)benzene (1k)[2]**:** yellow solid, 79% yield. **^{1}H NMR** (400 MHz, DMSO-d_6) δ: 8.74 (s, 1H); 8.43 (d, *J* = 13.7 Hz, 1H); 8.37 – 8.26 (m, 3H); 7.78 (t, *J* = 8.0 Hz, 1H).

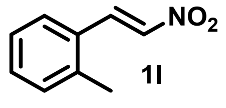

1l

(*E*)-1-methyl-2-(2-nitrovinyl)benzene (1l)[2]**:** yellow solid, 70% yield. **^{1}H NMR** (400 MHz, $CDCl_3$) δ: 8.28 (d, *J* = 13.6 Hz, 1H); 7.51(s, 1H); 7.50-7.47 (m, 1H); 7.42 – 7.34 (m, 1H); 7.30 – 7.23 (m, 2H); 2.47 (s, 3H).

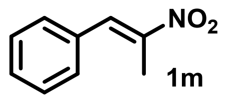

1m

(*E*)-(2-nitroprop-1-en-1-yl)benzene (4m)[3]**:** yellow solid, 72% yield. **^{1}H NMR** (400 MHz, $CDCl_3$) δ: 8.10 (s, 1H); 7.50 – 7.40 (m, 5H); 2.46 (d, *J* = 1.1 Hz, 3H).

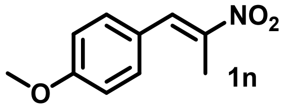

1n

(*E*)-1-methoxy-4-(2-nitroprop-1-en-1-yl)benzene (1n)[3]: yellow solid, 67% yield. **^{1}H NMR** (400 MHz, $CDCl_3$) δ: 8.06 (s, 1H); 7.45 – 7.38 (m, 2H); 7.00 – 6.94 (m, 2H); 3.85 (s, 3H); 2.46 (d, *J* = 1.0 Hz, 3H).

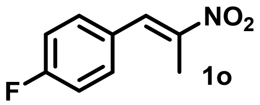

1o

(*E*)-1-fluoro-4-(2-nitroprop-1-en-1-yl)benzene (1o)[3]**:** yellow solid, 74% yield. **^{1}H NMR** (400 MHz, $CDCl_3$) δ: 8.05 (s, 1H); 7.48 – 7.40 (m, 2H); 7.20 – 7.12 (m, 2H); 2.45 (d, J = 0.9 Hz, 3H).

General microwave procedure for the synthesis of triazoles 2a-g

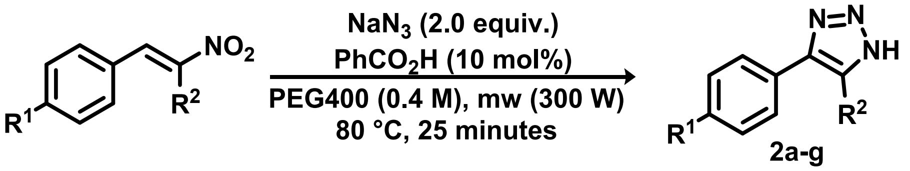

A 5 mL resealable microwave reaction vessel was charged with the corresponding nitroolefins (0.20 mmol), benzoic acid (12.2 mg, 50 mol%), sodium azide (26.0 mg, 0.4 mmol) and PEG400 (0.5 mL). The mixture was irradiated at 300 W with a constant temperature of 80 °C for 25 minutes. Then, the mixture was extracted with H_2O (15 mL) and EtOAc (3 x 10 mL) and the organic phase was dried with Na_2SO_4 and concentrated under vacuum. The crude products were purified via flash column chromatography utilizing a gradient of hexane/EtOAc mixture as eluent. The purified products were obtained with a hexane/EtOAc (8:2) proportion in all cases.

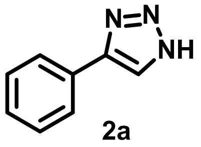

2a

4-phenyl-1*H*-1,2,3-triazole (2a)[4]**:** white solid, 62% yield. **^{1}H NMR** (400 MHz, DMSO-d_6) δ: 15.03 (s, 1H); 8.33 (s, 1H); 7.87 (d, J = 7.4 Hz, 2H); 7.47 (d, J = 7.3 Hz, 2H); 7.35 (d, J = 6.0 Hz, 1H).

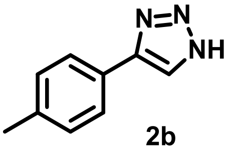

2b

4-(*p*-tolyl)-1*H*-1,2,3-triazole (2b)[4]**:** white solid, 31% yield. **^{1}H NMR** (400 MHz, $CDCl_3$) δ: 12.96 (s, 1H); 7.95 (s, 1H); 7.71 (d, J = 8.0 Hz, 2H); 7.30 – 7.22 (m, 2H); 2.38 (d, J = 6.4 Hz, 3H).

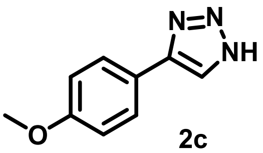

2c

4-(4-methoxyphenyl)-1*H*-1,2,3-triazole (1c)[4]: white solid, 34% yield. **^{1}H NMR** (400 MHz, $CDCl_3$) δ: 12.76 (s, 1H); 7.90 (s, 1H); 7.79 – 7.71 (m, 2H); 7.01 – 6.96 (m, 2H); 3.86 (s, 3H).

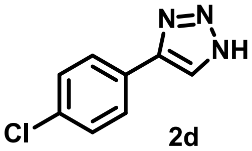

2d

4-(4-chlorophenyl)-1*H*-1,2,3-triazole (2d)[4]: white solid, 35% yield. **^{1}H NMR** (400 MHz, $CDCl_3$) δ: 12.64 (s, 1H); 7.96 (s, 1H); 7.79 – 7.74 (m, 2H); 7.45 – 7.41 (m, 2H).

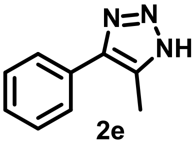

2e

5-methyl-4-phenyl-1*H*-1,2,3-triazole (2e)[4]: white solid, 57% yield. **^{1}H NMR** (400 MHz, $CDCl_3$) δ: 12.15 (s, 1H); 7.74 – 7.69 (m, 2H); 7.50 – 7.44 (m, 2H); 7.42 – 7.36 (m, 1H); 2.55 (s, 3H).

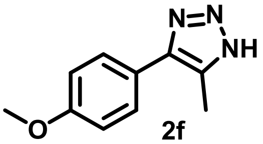

2f

4-(4-methoxyphenyl)-5-methyl-1*H*-1,2,3-triazole (2f)[4]: white solid, 35% yield. **^{1}H NMR** (400 MHz, $CDCl_3$) δ: 12.34 (s, 1H); 7.67 – 7.60 (m, 2H); 7.02 – 6.96 (m, 2H); 3.86 (s, 3H); 2.52 (s, 3H).

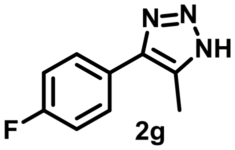

2g

4-(4-fluorophenyl)-5-methyl-1*H*-1,2,3-triazole (2g)[4]: white solid, 35% yield. **^{1}H NMR** (400 MHz, $CDCl_3$) δ: 12,34 (s, 1H); 7,67 – 7,60 (m, 2H); 7,02 – 6,96 (m, 2H); 3,86 (s, 3H); 2,52 (s, 3H).

Microwave sequential "one-pot" procedure for the synthesis of compounds 4a-o

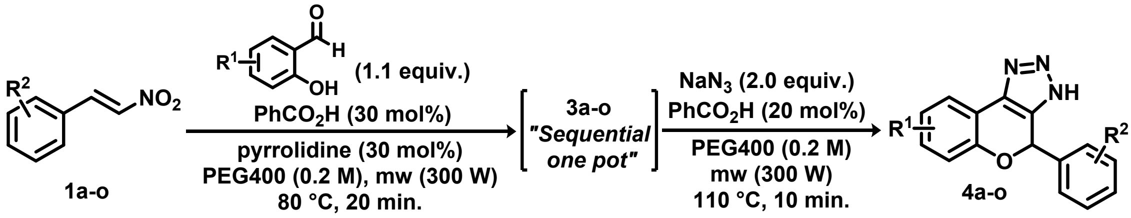

Step 1: A 5 mL resealable microwave reaction vessel was charged with the corresponding nitroolefins (0.20 mmol), substituted salicylaldehydes (0.22 mmol), benzoic acid (7.2 mg, 30 mol%), pyrrolidine (5.5 μL, 30 mol%) and PEG400 (1.0 mL). The mixture was irradiated at 300 W with a constant temperature of 110 °C for 20 minutes.

Step 2: Next, benzoic acid (4.8 mg, 20 mol%) and sodium azide (26.0 mg, 0.4 mmol) were added in sequence, and the mixture was irradiated (300 W) at 110 °C for 10 minutes. The mixture was extracted with H_2O (15 mL) and EtOAc (3 x 10 mL) and the organic phase was dried with Na_2SO_4 and concentrated under vacuum. The crude products were purified via flash column chromatography utilizing a gradient of hexane/EtOAc mixture as eluent. The purified products were obtained with a hexane/EtOAc (8:2) proportion in all cases.

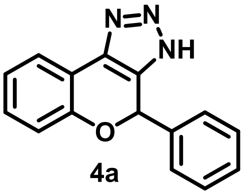

4a

4-phenyl-3,4-dihydrochromeno[3,4-*d*][1,2,3]triazole (4a)[5]: White solid, 80% yield. **^{1}H NMR** (400 MHz, DMSO-d$_6$) δ: 15.14 (s, 1H); 7.68 (d, *J* = 7.3 Hz, 1H); 7.39 – 7.35 (m, 5H); 7.30 – 7.25 (m, 1H); 7.09 – 7.03 (m, 2H); 6.77 (s, 1H). **^{13}C NMR** (100 MHz, DMSO-d$_6$) δ: 152.5; 139.1; 130.1; 128.6; 127.0; 122.6; 122.2; 117.4; 116.1; 75.4.

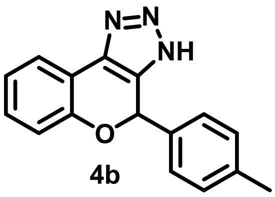

4b

4-(*p*-tolyl)-3,4-dihydrochromeno[3,4-*d*][1,2,3]triazole (4b)[5]: White solid, 57% yield. **^{1}H NMR** (400 MHz, $CDCl_3$) δ: 12.44 (s, 1H); 7.82 – 7.74 (m, 1H); 7.35 – 7.25 (m, 3H); 7.21 – 7.13 (m, 2H); 7.08 – 7.02 (m, 2H); 6.55 (s, 1H); 2.32 (s, 3H). **^{13}C NMR** (100 MHz,

$CDCl_3$) δ: 153.6; 142.4; 139.0; 135.7; 130.6; 129.6; 127.2; 123.4; 122.4; 117.9; 115.8; 76.1; 21.4.

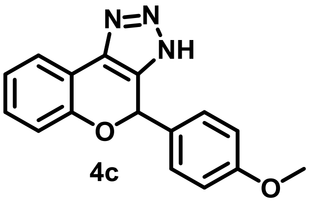

4c

4-(4-methoxyphenyl)-3,4-dihydrochromeno[3,4-*d*][1,2,3]triazole (4c)[5]: White solid, 54% yield. **^{1}H NMR** (400 MHz, $CDCl_3$) δ: 12.17 (s, 1H); 7.80 – 7.75 (m, 1H); 7.36 (d, J = 8.7 Hz, 2H); 7.30 – 7.27 (m, 1H); 7.08 – 7.02 (m, 2H); 6.93 – 6.87 (m, 2H); 6.53 (s, 1H); 3.79 (s, 3H). **^{13}C NMR** (100 MHz, $CDCl_3$) δ: 160.2; 153.7; 148.3; 139.2; 130.6; 128.9; 123.4; 122.4; 117.9; 115.9; 114.3; 76.0; 55.5.

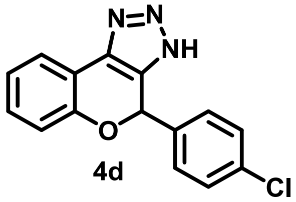

4d

4-(4-chlorophenyl)-3,4-dihydrochromeno[3,4-*d*][1,2,3]triazole (4d)[5]: White solid, 70% yield. **^{1}H NMR** (400 MHz, $CDCl_3$) δ: 11.92 (s, 1H); 7.77 (dd, J = 7.6, 1.7 Hz, 1H); 7.41 – 7.34 (m, 4H); 7.32 – 7.27 (m, 1H); 7.10 – 7.04 (m, 2H); 6.57 (s, 1H). **^{13}C NMR** (100 MHz, $CDCl_3$) δ: 151.1; 138.6; 129.4; 128.6; 128.5; 127.0; 126.0; 121.8; 119.1; 75.7.

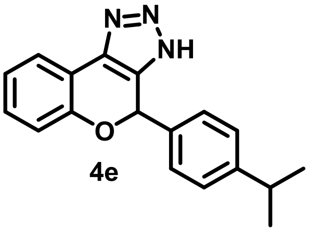

4e

4-(4-isopropylphenyl)-3,4-dihydrochromeno[3,4-*d*][1,2,3]triazole (4e): White solid. **Rf:** 0.40 (hexane/EtOAc 8:2), 37.8 mg, 0.13 mmol, 64% yield. **Melting point:** 144-146 °C. **^{1}H NMR** (400 MHz, $CDCl_3$) δ: 12.92 (s, 1H), 7.78 (dd, J = 7.8, 1.5 Hz, 1H), 7.36 (d, J = 8.2 Hz, 2H), 7.29 – 7.20 (m, 3H), 7.07 – 7.01 (m, 2H), 6.55 (s, 1H), 2.87 (sept, J = 7.1 Hz, 1H), 1.20 (d, J = 6.9 Hz, 6H). **^{13}C NMR** (100 MHz, $CDCl_3$) δ: 153.5, 149.7,

142.1, 138.7, 135.9, 130.5, 127.2, 126.9, 123.3, 122.3, 117.8, 115.6, 76.1, 33.9, 23.9. **HRMS (ESI-TOF)** m/z $[M+H]^+$ for $C_{18}H_{18}N_3O$: cald. – 292.1444 found - 292.1444.

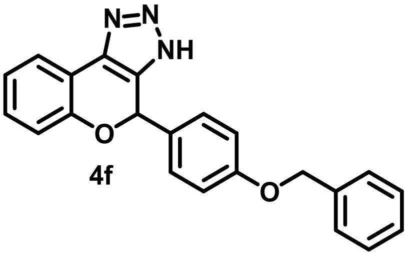

4f

4-(4-(benzyloxy)phenyl)-3,4-dihydrochromeno[3,4-*d*][1,2,3]triazole (4f): White solid. **Rf**: 0.31 (hexane/EtOAc 8:2), (43.3 mg, 0.12 mmol), 61% yield. **Melting point:** 172-174 °C. **^{1}H NMR** (400 MHz, $CDCl_3$) δ: 11.74 (s, 1H), 7.77 (dd, *J* = 7.7, 1.6 Hz, 1H), 7.45 – 7.33 (m, 7H), 7.26 (s, 1H), 7.08 – 7.02 (m, 2H), 6.99 – 6.95 (m, 2H), 6.53 (s, 1H), 5.05 (s, 2H). **^{13}C NMR** (100 MHz, $CDCl_3$) δ: 159.3, 157.3, 153.5, 142.5, 136.8, 133.8, 130.9, 130.5, 128.7, 128.6, 128.0, 127.4, 123.2, 122.3, 117.8, 115.7, 115.0, 75.9, 70.1. **HRMS (ESI-TOF)** m/z $[M+H]^+$ for $C_{22}H_{18}N_3O_2$: cald. – 356.1393 found – 356.1392.

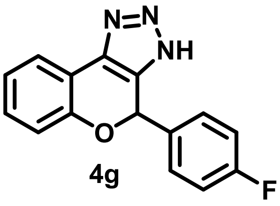

4g

4-(4-fluorophenyl)-3,4-dihydrochromeno[3,4-*d*][1,2,3]triazole (4g): Off-white solid. **Rf**: 0.32 (hexane/EtOAc 8:2), 36.8 mg, 0.13 mmol, 69% yield. **Melting point:** 147-149 °C. **^{1}H NMR** (400 MHz, DMSO) δ: 15.20 (s, 1H), 7.69 (d, *J* = 7.1 Hz, 1H), 7.43 (dd, *J* = 8.5, 5.7 Hz, 2H), 7.29 (t, *J* = 8.6 Hz, 2H), 7.23 (t, *J* = 8.8 Hz, 2H), 7.12 – 7.03 (m, 2H), 6.80 (s, 1H). **^{13}C NMR** (100 MHz, DMSO) δ: 163.3, 160.9, 152.3, 137.0, 135.2, 130.1, 129.4 (d, *J* = 8.6 Hz), 122.7, 122.6, 122.3, 118.2, 117.4, 115.9, 115.6 (d, *J* = 21.9 Hz), 74.7. **HRMS (ESI-TOF)** m/z $[M+H]^+$ for $C_{15}H_{11}FN_3O$: cald. – 268.0880 found – 268.0881.

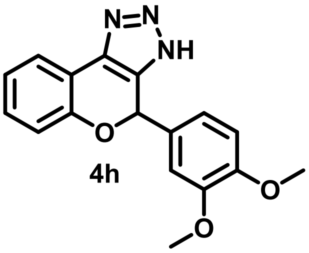

4h

4-(3,4-dimethoxyphenyl)-3,4-dihydrochromeno[3,4-*d*][1,2,3]triazole (4h): White solid. **Rf**: 0.36 (hexane/EtOAc 8:2), 34.6 mg, 0.11 mmol, 56% yield. **Melting point:** 183-185 °C. **^{1}H NMR** (400 MHz, $CDCl_3$) δ: 11.92 (s, 1H); 7.78 (dd, *J* = 7.5, 1.4 Hz, 1H); 7.31 – 7.28 (m, 1H); 7.11 – 7.03 (m, 2H); 7.01 – 6.95 (m, 2H); 6.87 – 6.84 (m, 1H); 6.52 (s, 1H); 3.86 (d, *J* = 10.5 Hz, 6H). **^{13}C NMR** (100 MHz, $CDCl_3$) δ: 153.7; 149.7; 149.4; 131.0; 130.7; 123.4; 122.5; 120.1; 117.9; 115.9; 111.2; 110.5; 76.3; 56.0. **HRMS (ESI-TOF)** m/z $[M+H]^+$ for $C_{17}H_{16}N_3O_3$: cald. – 310.1186 found – 310.1183.

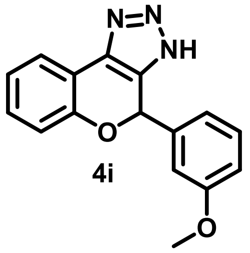

4i

4-(3-methoxyphenyl)-3,4-dihydrochromeno[3,4-*d*][1,2,3]triazole (4i): White solid. **Rf**: 0.41 (hexane/EtOAc 8:2), 33.5 mg, 0.12 mmol, 60% yield. **Melting point:** 126-128 °C. **^{1}H NMR** (400 MHz, $CDCl_3$) δ: 12.79 (s, 1H), 7.78 (dd, *J* = 7.5, 1.3 Hz, 1H), 7.30 – 7.25 (m, 2H), 7.10 – 6.98 (m, 4H), 6.86 (ddd, *J* = 8.3, 2.6, 0.9 Hz, 1H), 6.57 (s, 1H), 3.75 (s, 3H).**^{13}C NMR** (100 MHz, $CDCl_3$) δ: 159.8, 153.4, 141.9, 140.0, 138.7, 130.6, 129.8, 123.3, 122.4, 119.3, 117.8, 115.6, 114.4, 112.6, 75.9, 55.3. **HRMS (ESI-TOF)** m/z $[M+H]^+$ for $C_{16}H_{14}N_3O_2$: cald. – 280.1080 found – 280.1083.

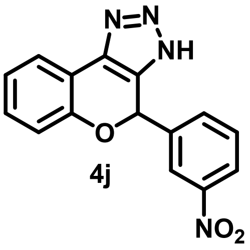

4j

4-(3-nitrophenyl)-3,4-dihydrochromeno[3,4-d][1,2,3]triazole (4j): White solid. **Rf**: 0.36 (hexane/EtOAc 8:2), 38.8 mg, 0.13 mmol, 66% yield. **Melting point:** 188-190 °C. **^{1}H NMR** (400 MHz, $CDCl_3$) δ: 12.38 (s, 1H), 8.38 (t, *J* = 2.0 Hz, 1H), 8.21 (ddd, *J* = 8.2, 2.2, 1.0 Hz, 1H), 7.84 (dt, *J* = 7.4, 0.6 Hz, 1H), 7.79 (dd, *J* = 8.0, 1.7 Hz, 1H), 7.57 (t, *J* = 8.0 Hz, 1H), 7.37 – 7.28 (m, 1H), 7.12 (d, *J* = 8.0 Hz, 2H), 6.69 (s, 1H). **^{13}C NMR** (100 MHz, $CDCl_3$) δ: 152.9, 148.5, 141.2, 140.6, 133.0, 131.0, 129.7, 123.7, 123.4, 122.9, 122.1, 117.8, 115.4, 74.7. **HRMS (ESI-TOF)** m/z $[M+H]^+$ for $C_{15}H_{11}N_4O_3$: cald. – 295.08256 found - 295.08257.

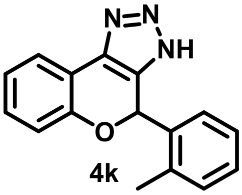

4-(*o*-tolyl)-3,4-dihydrochromeno[3,4-*d*][1,2,3]triazole (4k): White solid. **Rf**: 0.30 (hexane/EtOAc 8:2), 32.0 mg, 0.12 mmol, 61% yield. **Melting point:** 155-157 °C. **^{1}H NMR** (400 MHz, $CDCl_3$) δ: 12.68 (s, 1H), 7.80 (dd, *J* = 7.6, 1.6 Hz, 1H), 7.27 – 7.22 (m, 4H), 7.21 – 7.11 (m, 1H), 7.06 (td, *J* = 7.5, 1.1 Hz, 1H), 7.01 (dd, *J* = 8.2, 1.0 Hz, 1H), 6.75 (s, 1H), 2.48 (s, 3H). **^{13}C NMR** (100 MHz, $CDCl_3$) δ: 153.8, 141.9, 139.3, 136.5, 136.3, 131.0, 130.5, 129.0, 127.7, 126.2, 123.3, 122.3, 117.6, 115.6, 74.2, 19.4. **HRMS (ESI-TOF)** m/z $[M+H]^+$ for $C_{16}H_{14}N_3O$: cald. – 264.1131 found - 264,1129.

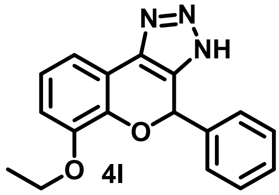

6-ethoxy-4-phenyl-3,4-dihydrochromeno[3,4-*d*][1,2,3]triazole (4l): White solid. **Rf**: 0.46 (hexane/EtOAc 8:2), 34.0 mg, 0.12 mmol, 58% yield. **Melting point:** 129-131 °C. **^{1}H NMR** (400 MHz, $CDCl_3$) δ: 11.96 (s, 1H); 7.49 – 7.44 (m, 2H); 7.39 – 7.28 (m, 4H); 7.01 – 6.96 (m, 1H); 6.92 (dd, *J* = 8.2, 1.5 Hz, 1H); 6.72 (s, 1H); 4.18 – 4.10 (m, 2H); 1.44 (t, *J* = 7.0 Hz, 3H). **^{13}C NMR** (100 MHz, $CDCl_3$) δ: 148.6; 143.0; 142.4; 138.7; 128.7; 126.7; 122.4; 116.9; 115.5; 115.1; 75.7; 65.0; 15.0. **HRMS (ESI-TOF)** m/z $[M+H]^+$ for $C_{17}H_{16}N_3O_2$: cald. – 294.1237 found - 294.1235.

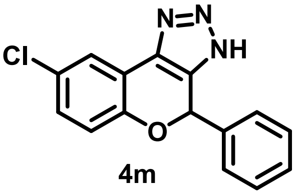

8-chloro-4-phenyl-3,4-dihydrochromeno[3,4-*d*][1,2,3]triazole (4m)[5]: White solid, 62% yield. **^{1}H NMR** (400 MHz, DMSO) δ: 15.28 (s, 1H); 7.64 (d, *J* = 2.6 Hz, 1H); 7.40 – 7.31 (m, 5H); 7.26 (dd, *J* = 8.7, 2.6 Hz, 1H); 7.04 (d, *J* = 8.7 Hz, 1H); 6.76 (s, 1H). **^{13}C NMR** (100 MHz, DMSO) δ: 151.1; 138.6; 129.4; 128.6; 128.5; 126.9; 125.9; 121.8; 119.1; 75.7.

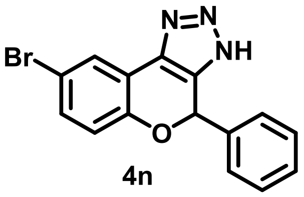

4n

8-bromo-4-phenyl-3,4-dihydrochromeno[3,4-*d*][1,2,3]triazole (4n): White solid. **Rf**: 0.43 (hexane/EtOAc 8:2, 36.0 mg, 0.11 mmol, 55% yield. **Melting point:** 162-164 °C. **^{1}H NMR** (400 MHz, DMSO) δ: 15.35 (s, 1H); 7.79 (d, *J* = 2.5 Hz, 1H); 7.45 – 7.41 (m, 1H); 7.41 – 7.33 (m, 5H); 7.02 (d, *J* = 8.7 Hz, 1H); 6.82 (s, 1H). **^{13}C NMR** (100 MHz, DMSO) δ: 151.6; 138.7; 132.5; 128.8; 128.7; 127.1; 124.7; 119.8; 113.6; 75.7. **HRMS (ESI-TOF)** m/z [M+H]$^+$ for $C_{15}H_{11}BrN_3O$: cald. – 328.0080 found – 328.0079.

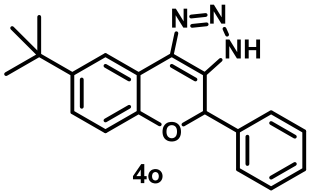

4o

8-(tert-butyl)-4-phenyl-3,4-dihydrochromeno[3,4-*d*][1,2,3]triazole (4o): White solid. **Rf**: 0.42 (hexane/EtOAc 8:2), 39.0 mg, 0.13 mmol, 64% yield. **Melting point:** 139-141 °C. **^{1}H NMR** (400 MHz, $CDCl_3$) δ: 13.33 (s, 1H); 7.89 (d, *J* = 2.4 Hz, 1H); 7.51 – 7.46 (m, 2H); 7.39 – 7.32 (m, 4H); 7.02 (d, *J* = 8.6 Hz, 1H); 6.59 (s, 1H); 1.35 (s, 9H). **^{13}C NMR** (100 MHz, $CDCl_3$) δ: 151.4; 145.5; 142.4; 139.3; 138.8; 128.9; 128.8; 127.8; 127.2; 120.3; 117.3; 115.0; 76.1; 34.6; 31.6. **HRMS (ESI-TOF)** m/z [M+H]$^+$ for $C_{19}H_{20}N_3O$: cald. – 306.1600 found – 306.1599.

General procedure for the three step "one-pot" microwave assisted synthesis of 4a, 4d and 4i

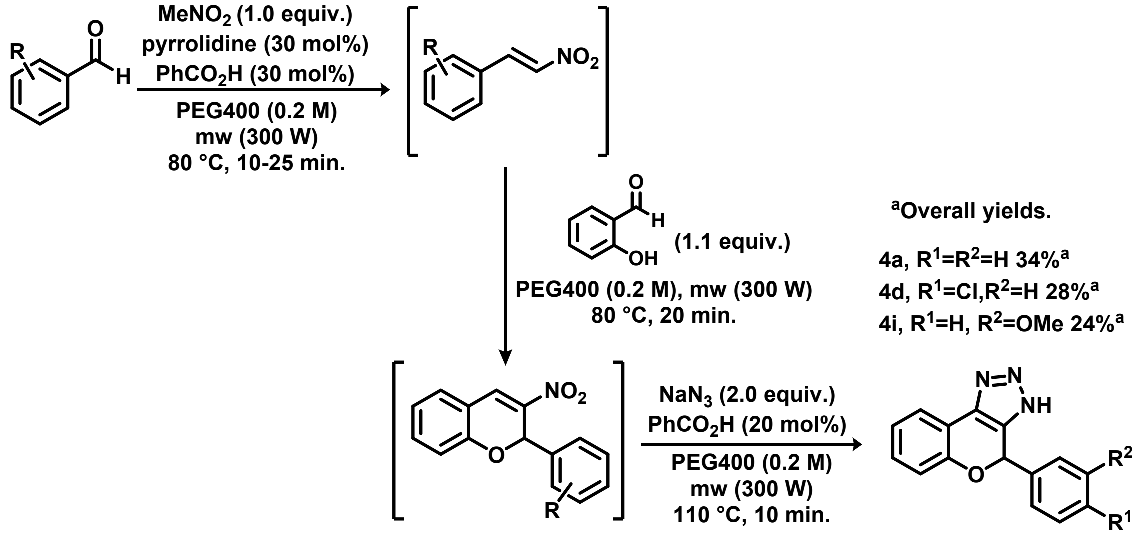

Step 1: A 5.0 mL resealable microwave reaction vessel was charged with the corresponding benzaldehyde derivatives (0.20 mmol), nitromethane (11 μL, 1.0 equiv.), benzoic acid (7.2 mg, 30 mol%), pyrrolidine (5.5 μL, 30 mol%) and PEG400 (1.0 mL). The mixture was irradiated at 300 W with a constant temperature of 80 °C for 10 - 25 minutes.

Step 2: Next, substituted salicylaldehydes (0.22 mmol) were added, and the mixture was irradiated at 300 W with a constant temperature of 80 °C for 20 minutes.

Step 3: Next, benzoic acid (4.8 mg, 20 mol%) and sodium azide (26.0 mg, 0.4 mmol) were added in sequence, and the mixture was irradiated (300 W) at 110 °C for 10 minutes. The mixture was extracted with H_2O (15 mL) and EtOAc (3 x 10 mL) and the organic phase was dried with Na_2SO_4 and concentrated under vacuum. The crude products were purified via flash column chromatography utilizing a gradient of hexane/EtOAc mixture as eluent. The purified products were obtained with a hexane/EtOAc (8:2) proportion in all cases.

Green Chemistry Metrics

Three metrics were selected and calculated according to the reported equations:[6,7]

$$\text{E-factor} = \frac{\text{mass of total waste}}{\text{mass of product}} \quad (1)$$

$$\text{Atom economy} = \frac{\text{molecular mass of desired product}}{\text{molecular masses of reactants}} \times 100\% \quad (2)$$

E-Factor: parameters used for the calculations of E-factor were showed in table 1 and 2

Table 1. Parameters for the calculation of E-factor for the multistep procedure with conventional heating.

Multistep procedure with conventional heating – TOTAL = 700.4758									
Solvents and Reactants	Synthesis of 1a	Synthesis of 3a	Synthesis of 4a	Extraction			Purification		
				1a	3a	4a	1a	3a	4a
1a	-	29.8 mg	-	-	-	-	-	-	-
3a	-	-	50,6 mg	-	-	-	-	-	-
PEG400	1.13 g	1.13 g	1.13 g	-	-	-	-	-	-
benzaldehyde	21.2 mg	-	-	-	-	-	-	-	-
nitromethane	12.2 mg	-	-	-	-	-	-	-	-
benzoic acid	7.2 mg	-	4.8 mg	-	-	-	-	-	-
pyrrolidine	4.7 mg	-	-	-	-	-	-	-	-
salicylaldehyde	-	29.3 mg	-	-	-	-	-	-	-
sodium azide	-	-	26.0 mg	-	-	-	-	-	-
Hexane (g)	-	-	-	-	-	-	-	163.8	163.8
ethyl acetate (g)	-	-	-	-	36	36	-	90.8	90.8
deionized H_2O (g)	-	-	-	5.0	5.0	5.0	-	-	-
EtOH (g)	-	-	-	-	-	-	4.7	-	-
Na_2SO_4 (g)	-	-	-	-	-	6.0	-	-	-
silica flash (g)	-	-	-	-	-	-	-	45	45
**TOTAL (g)**	1.1753	1.1891	1.2114	5	41	47	4.7	299.6	299.6

$$\text{E-factor (with extraction and purification)} = \frac{700.4758 - 0.0170}{0,0170} = 41203$$

$$\text{E-factor (without extraction and purification)} = \frac{3.3758 - 0.0170}{0.0170} = 198$$

Table 2. Parameters for the calculation of E-factor for the three step "one pot" procedure with microwave irradiation.

Three step "one pot" procedure with microwave irradiation – TOTAL = 347.8354									
**Solvents and Reactants**	**Synthesis of 1a**	**Synthesis of 3a**	**Synthesis of 4a**	**Extraction**			**Purification**		
				**1a**	**3a**	**4a**	**1a**	**3a**	**4a**
**1a**	-	-	-	-	-	-	-	-	-
**3a**	-	-	-	-	-	-	-	-	-
**PEG400**	1.13 g	-	-	-	-	-	-	-	-
**benzaldehyde**	21.2 mg	-	-	-	-	-	-	-	-
**nitromethane**	12.2 mg	-	-	-	-	-	-	-	-
**benzoic acid**	7.2 mg	-	4.8 mg	-	-	-	-	-	-
**pyrrolidine**	4.7 mg	-	-	-	-	-	-	-	-
**salicylaldehyde**	-	29.3 mg	-	-	-	-	-	-	-
**sodium azide**	-	-	26.0 mg	-	-	-	-	-	-
**Hexane (g)**	-	-	-	-	-	-	-	-	163.8
**ethyl acetate (g)**	-	-	-	-	-	36	-	-	90.8
**deionized H_2O (g)**	-	-	-	-	-	5.0	-	-	-
**EtOH (g)**	-	-	-	-	-	-	-	-	-
**Na_2SO_4 (g)**	-	-	-	-	-	6.0	-	-	-
**silica flash (g)**	-	-	-	-	-	-	-	-	45
**TOTAL (g)**	1.1753	0.0293	0.0308	-	-	47	-	-	299.6

$$\text{E-factor (with extraction and purification)} = \frac{347.8354 - 0.0170}{0.0170} = 20459$$

$$\text{E-factor (without extraction and purification)} = \frac{1.2354 - 0.0170}{0.0170} = 72$$

Atom economy: multistep procedure with conventional heating and three step "one pot" procedure with microwave irradiation

$$\text{Atom economy} = \frac{249{,}28}{106.12 + 61.04 + 122.12 + 65.00} \times 100\% = 70\%$$

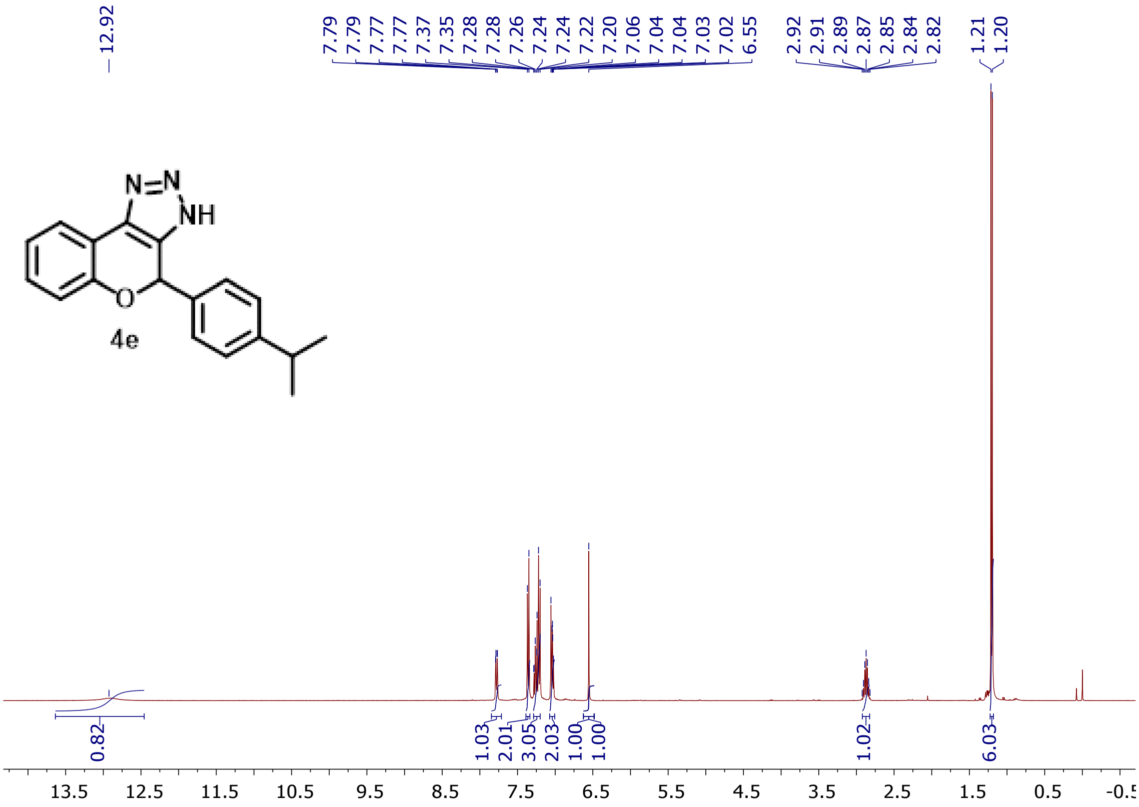

Figure 1. ^{1}H NMR spectra (400 MHz, $CDCl_3$) of compound **4e**.

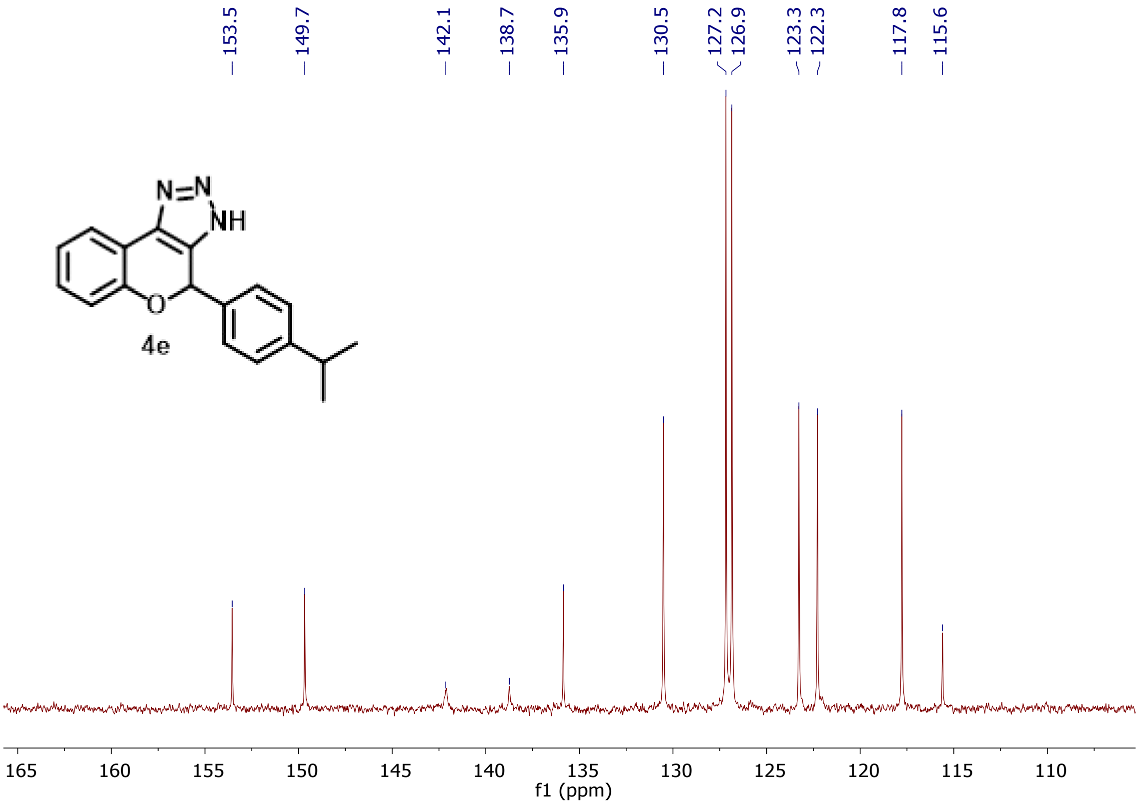

Figure 2. ^{13}C NMR spectra (100 MHz, $CDCl_3$) of compound **4e**.

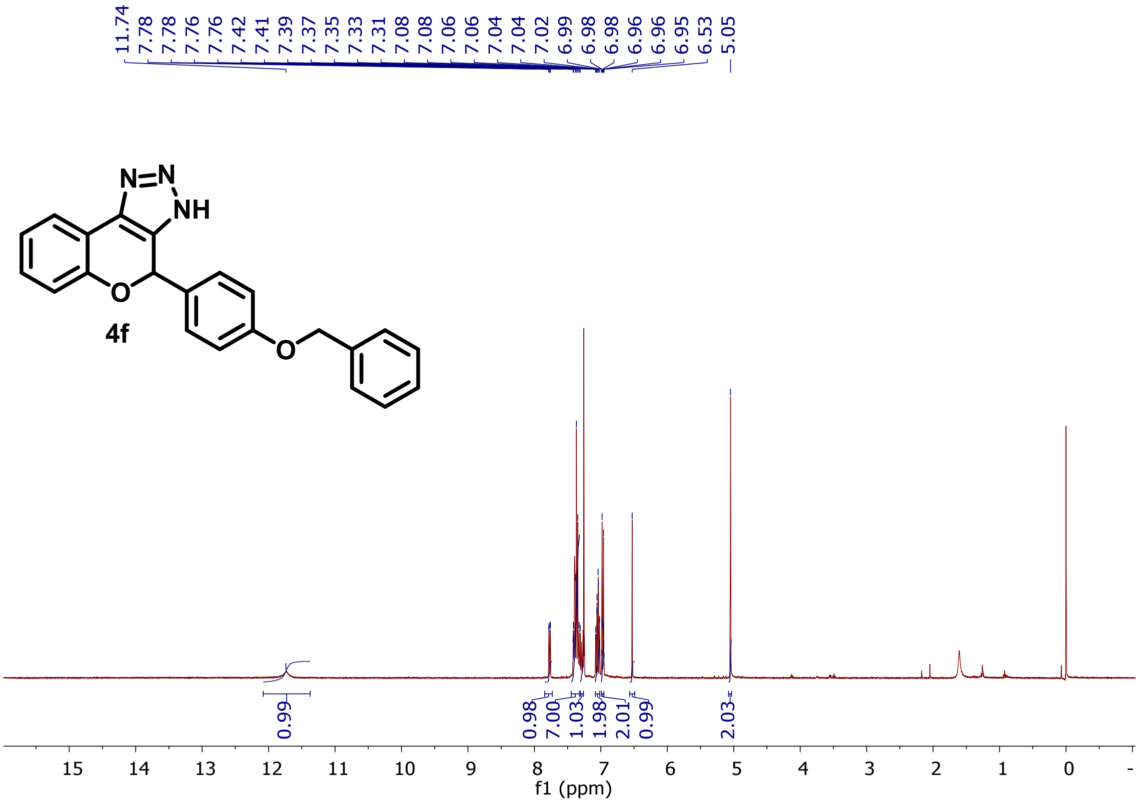

Figure 3. ^{1}H NMR spectra (400 MHz, $CDCl_3$) of compound **4f**.

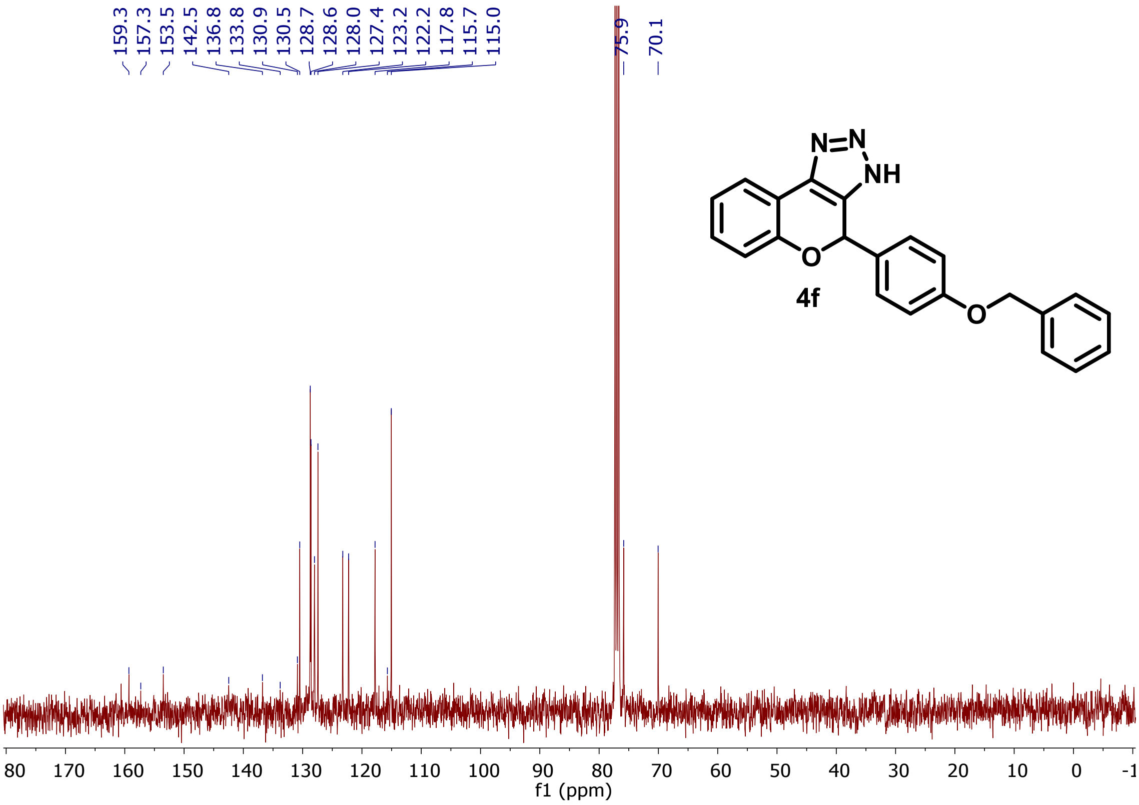

Figure 4. ^{13}C NMR spectra (100 MHz, $CDCl_3$) of compound **4f**.

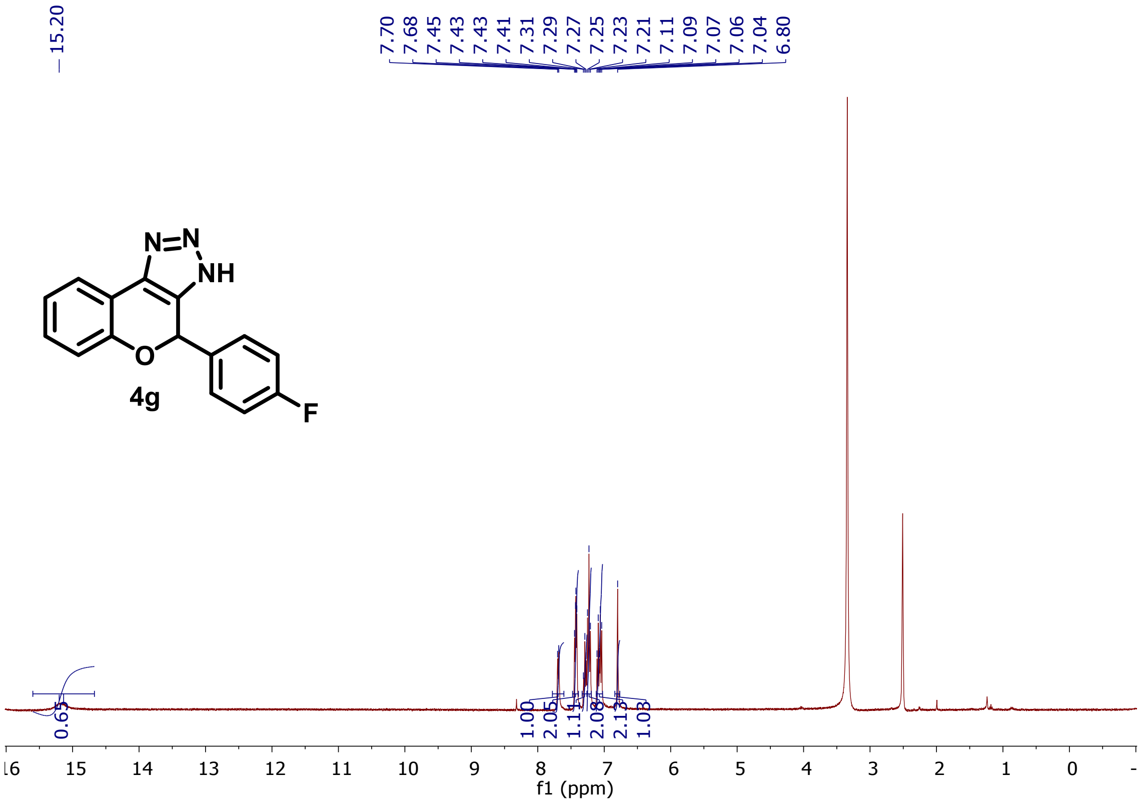

Figure 5. ^{1}H NMR spectra (400 MHz, DMSO-d_6) of compound **4g**.

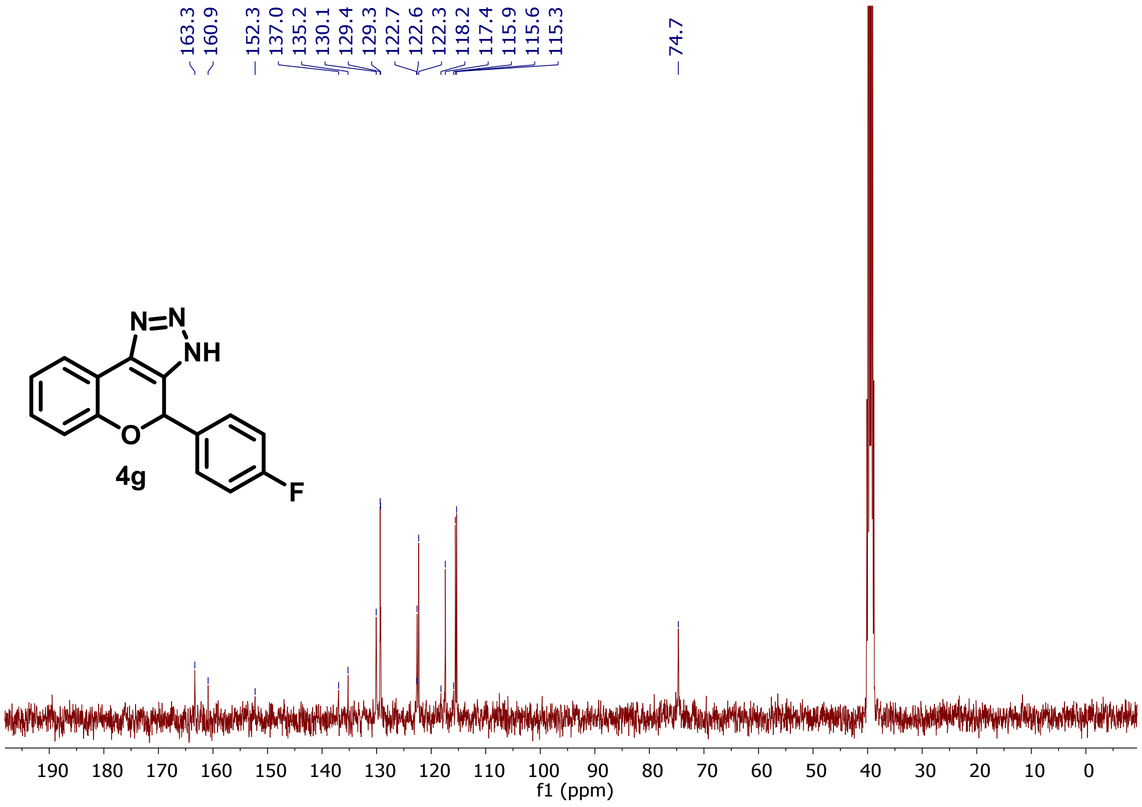

Figure 6. ^{13}C NMR spectra (100 MHz, DMSO-d_6) of compound **4g**.

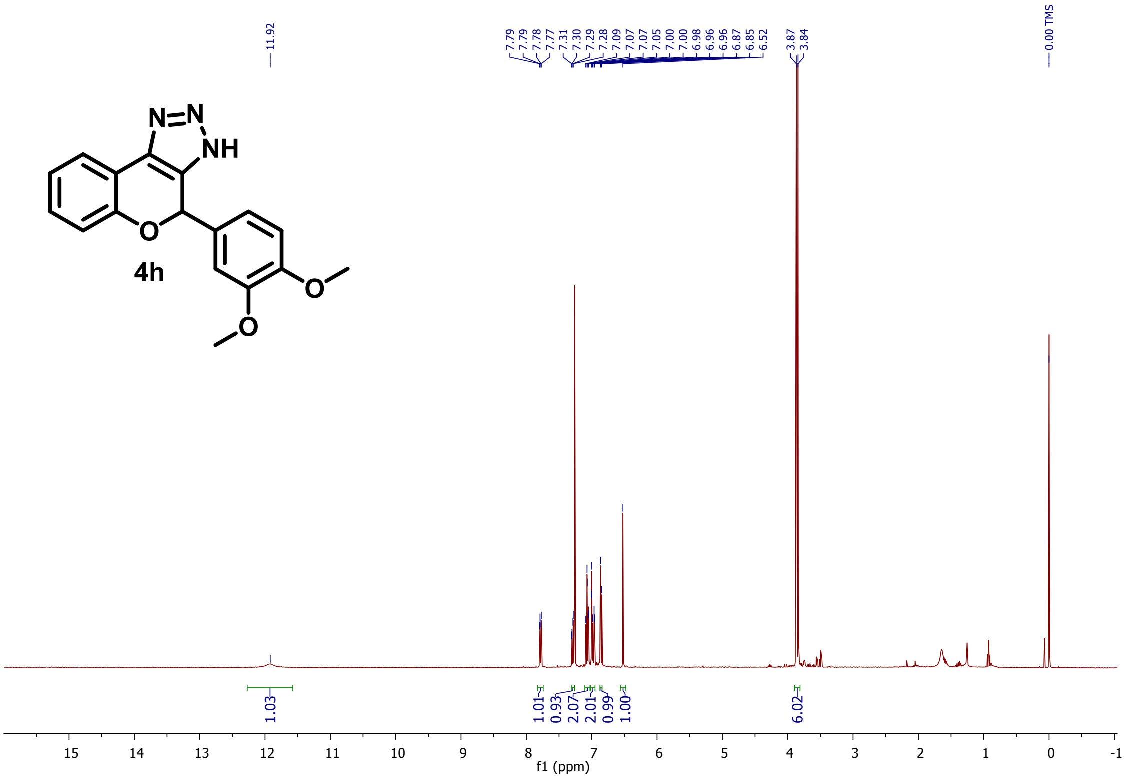

Figure 7. ^{1}H NMR spectra (400 MHz, $CDCl_3$) of compound **4h**.

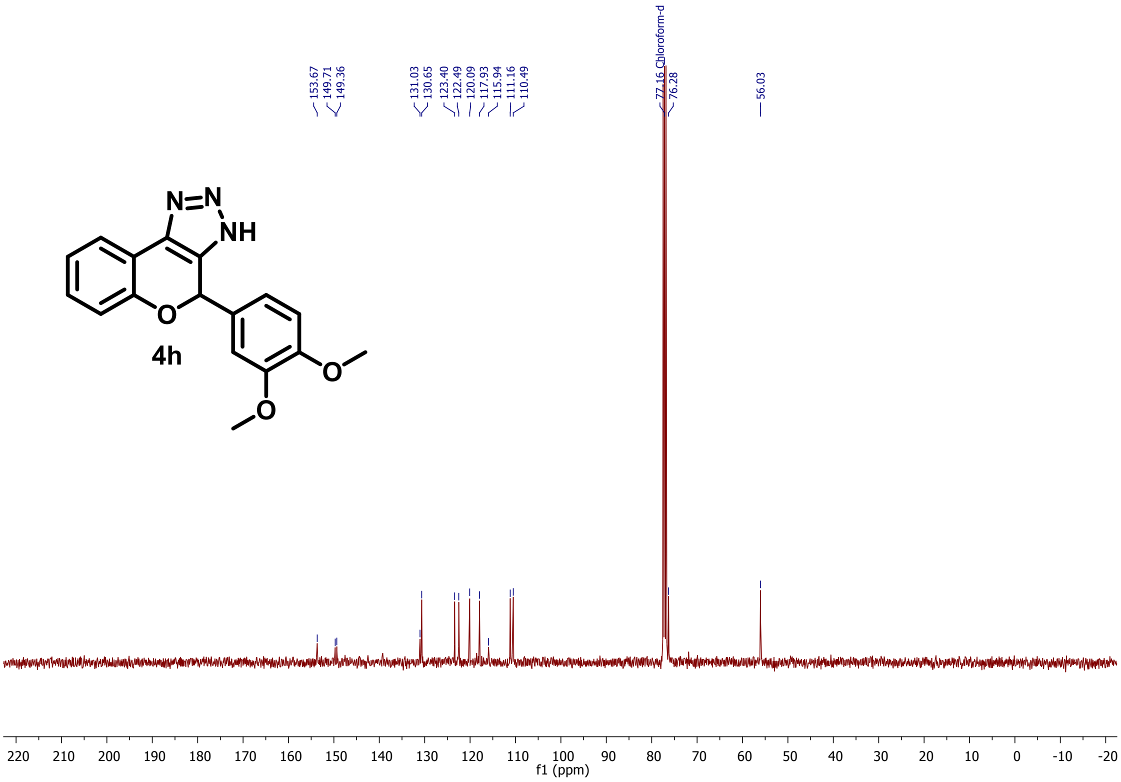

Figure 8. ^{13}C NMR spectra (100 MHz, $CDCl_3$) of compound **4h**.

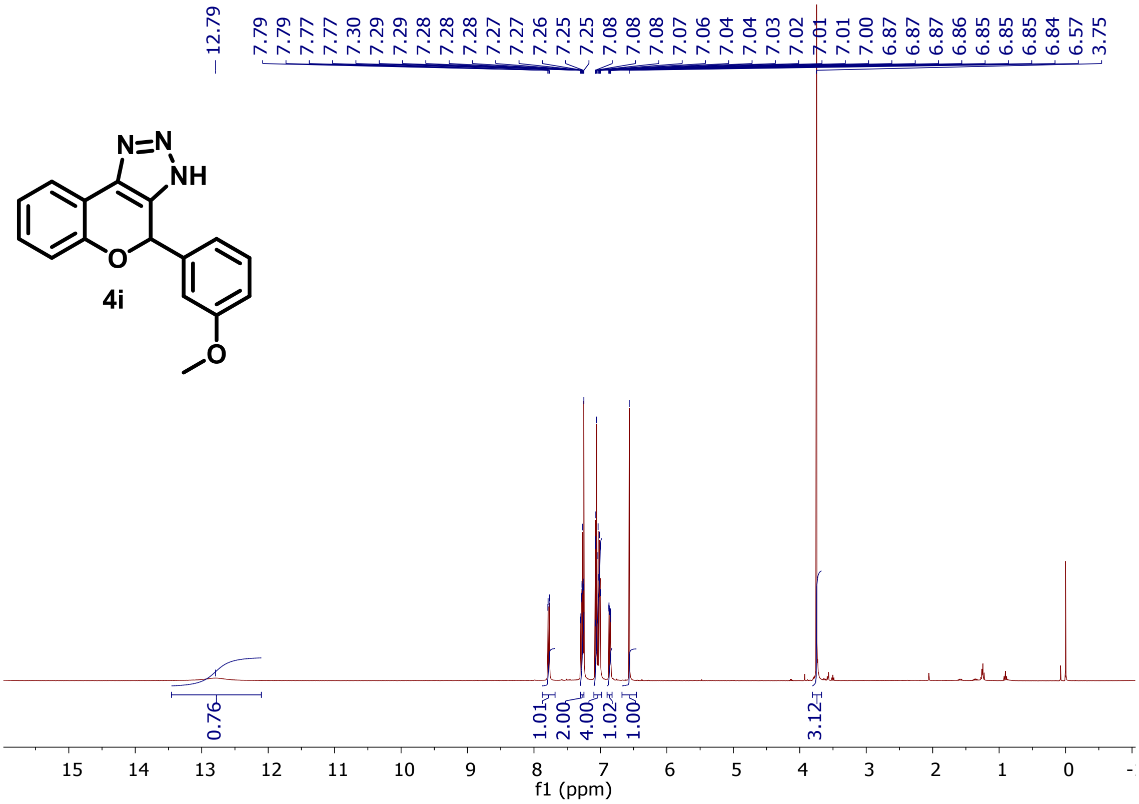

Figure 9. ^{1}H NMR spectra (400 MHz, $CDCl_3$) of compound **4i**.

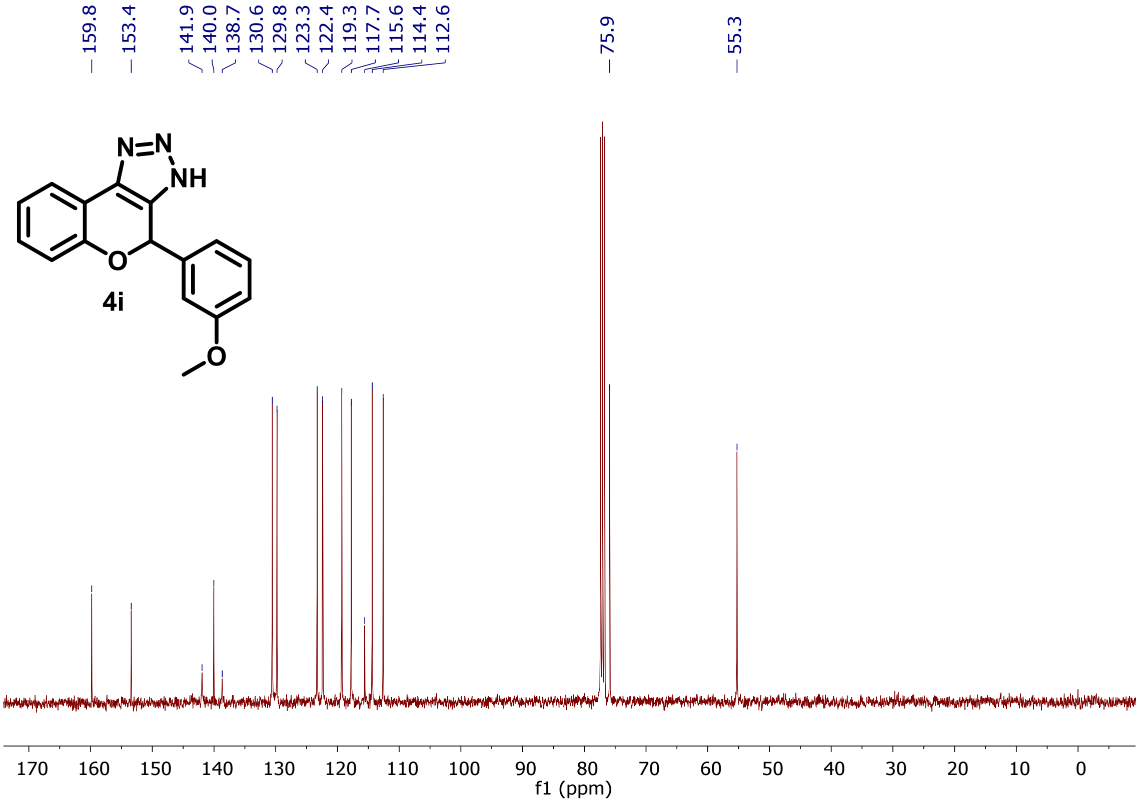

Figure 10. ^{13}C NMR spectra (100 MHz, $CDCl_3$) of compound **4i**.

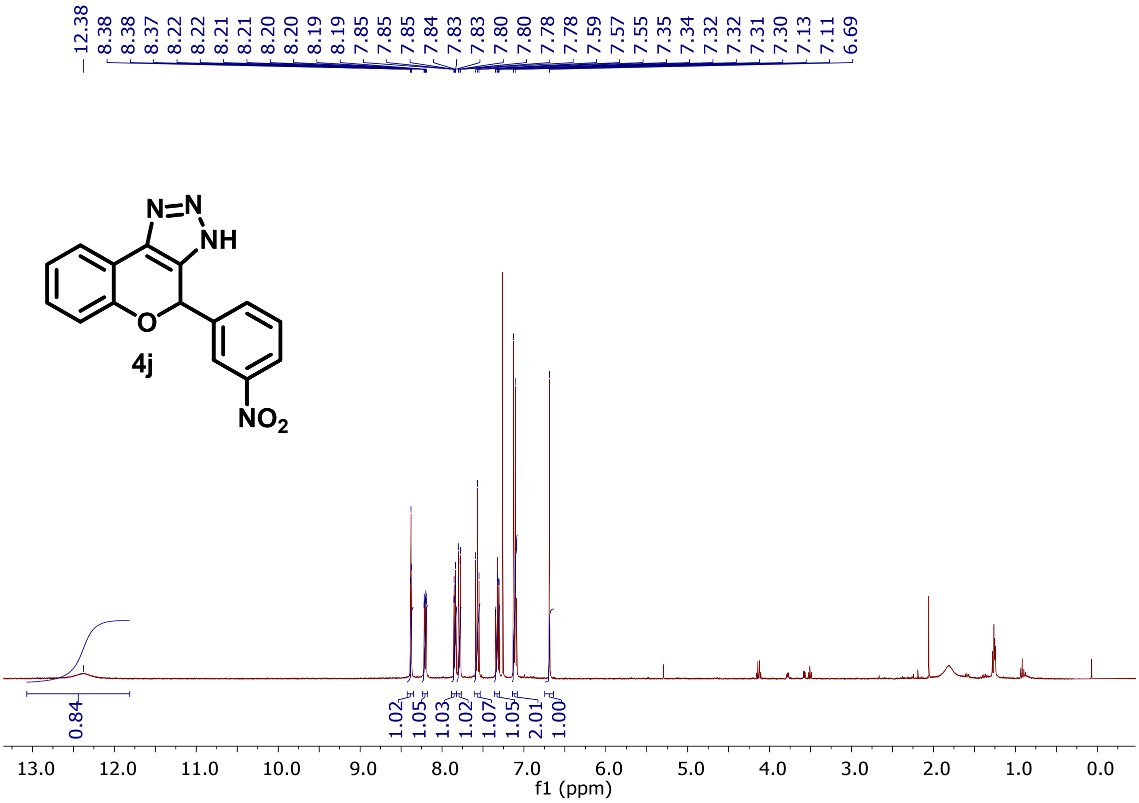

Figure 11. ^{1}H NMR spectra (400 MHz, $CDCl_3$) of compound **4j**.

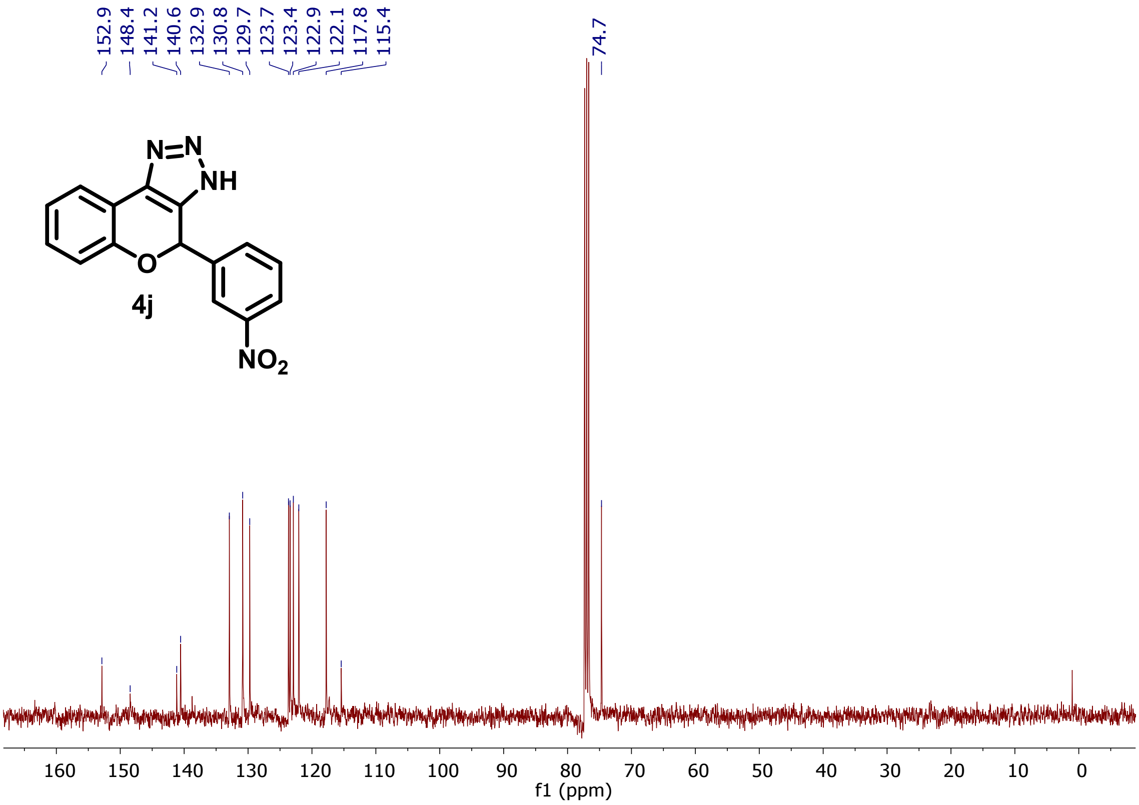

Figure 12. ^{13}C NMR spectra (100 MHz, $CDCl_3$) of compound **4j**.

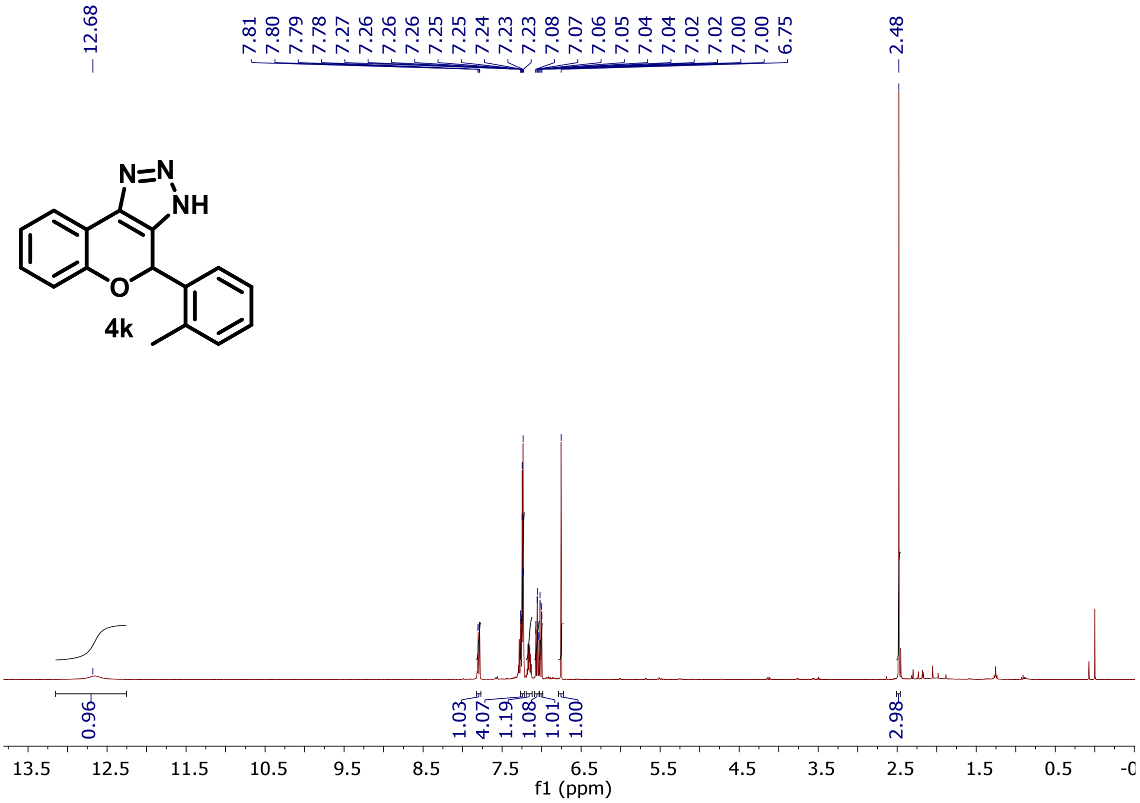

Figure 13. ^{1}H NMR spectra (400 MHz, $CDCl_3$) of compound **4k**.

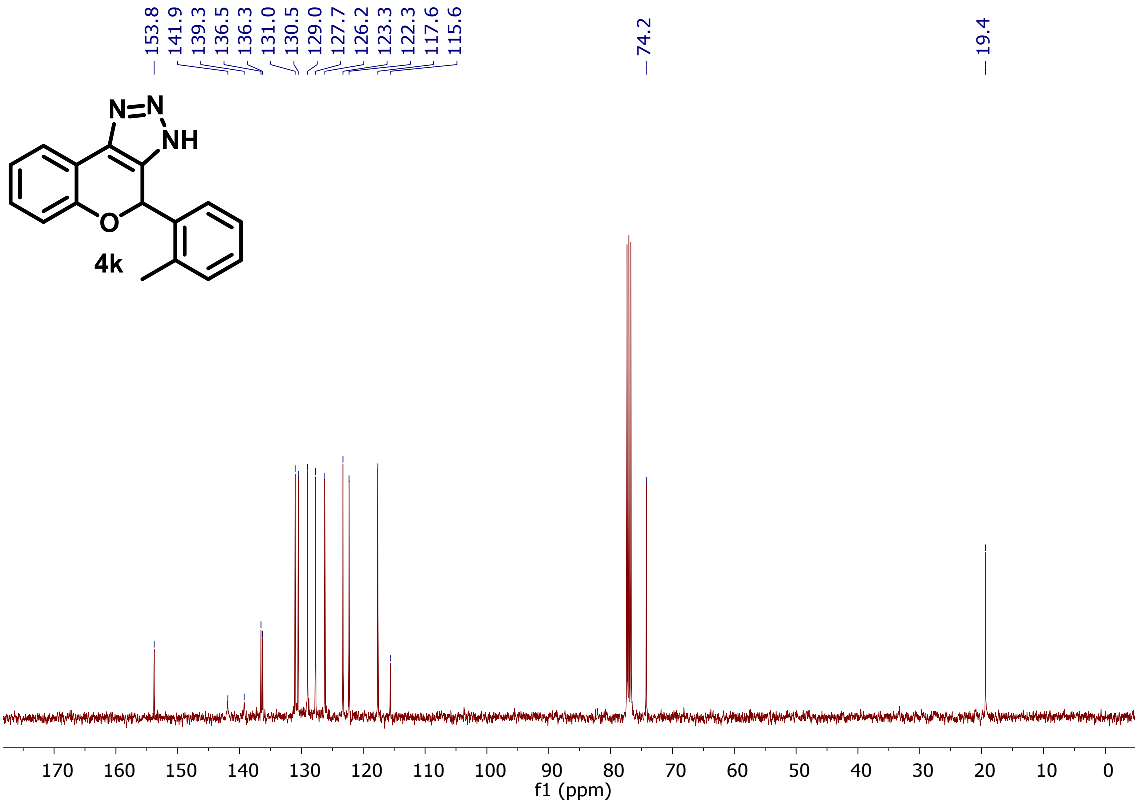

Figure 14. ^{13}C NMR spectra (100 MHz, $CDCl_3$) of compound **4k**.

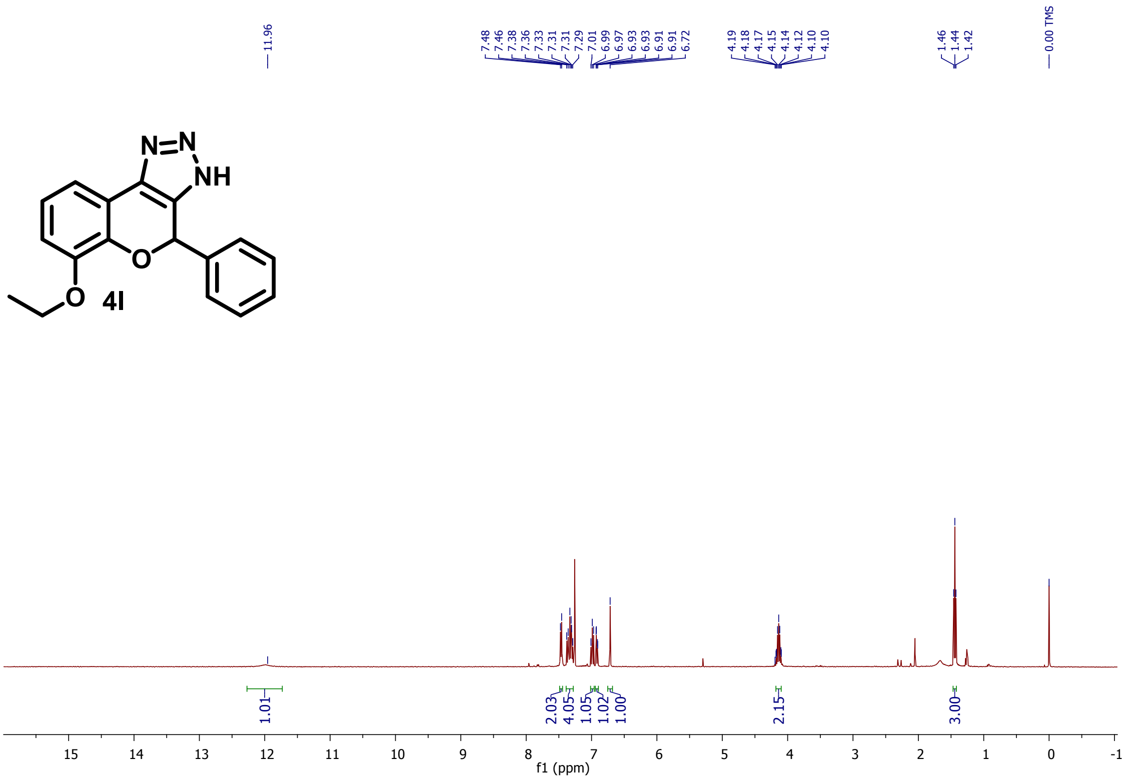

Figure 15. ^{1}H NMR spectra (400 MHz, $CDCl_3$) of compound **4l**.

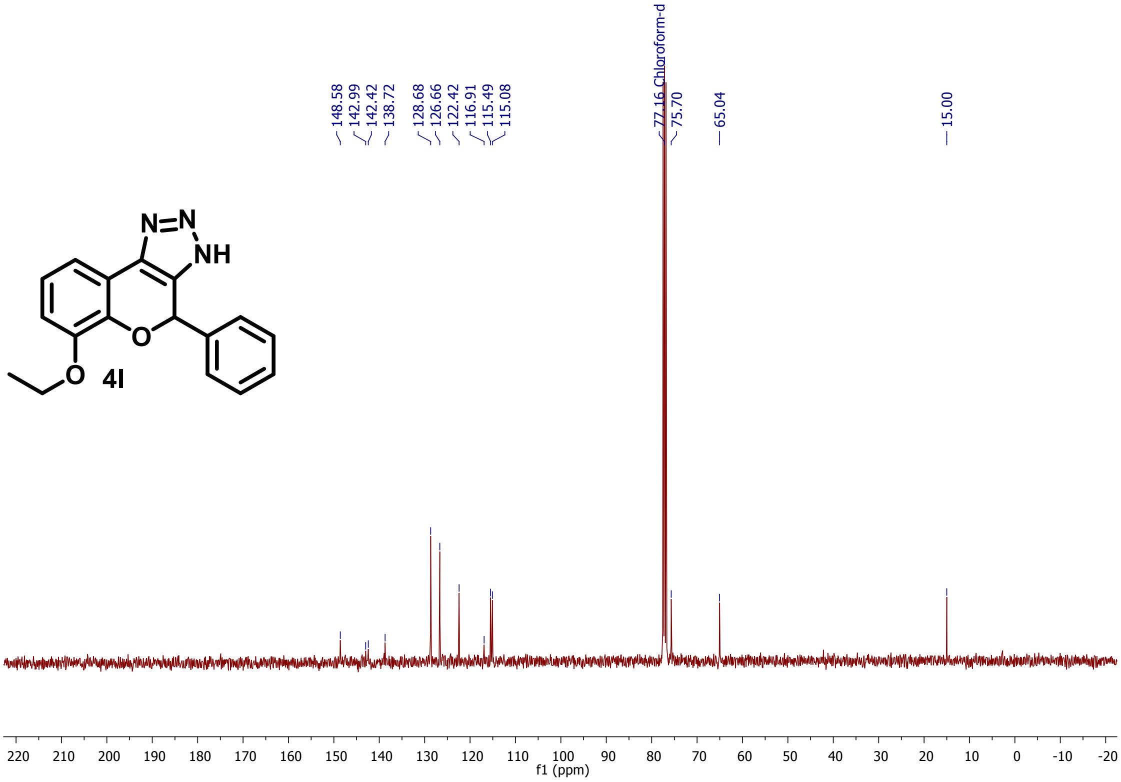

Figure 16. ^{13}C NMR spectra (100 MHz, $CDCl_3$) of compound **4l**.

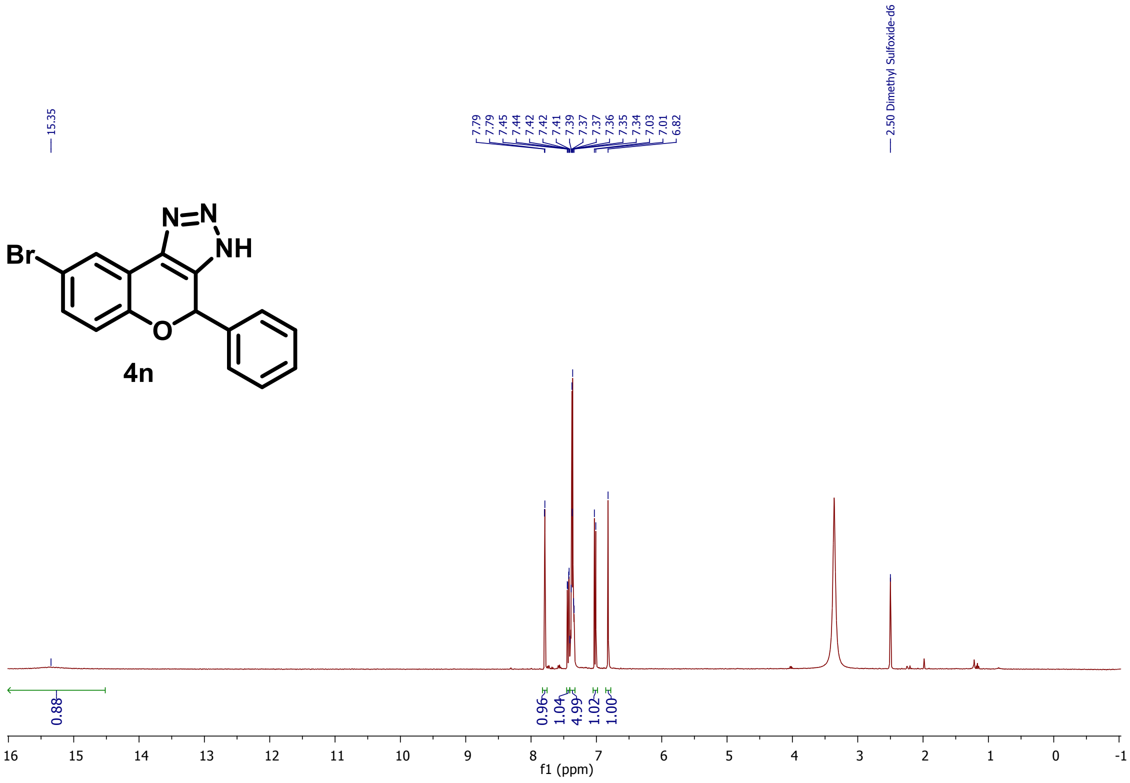

Figure 17. ^{1}H NMR spectra (400 MHz, DMSO-d_6) of compound **4n**.

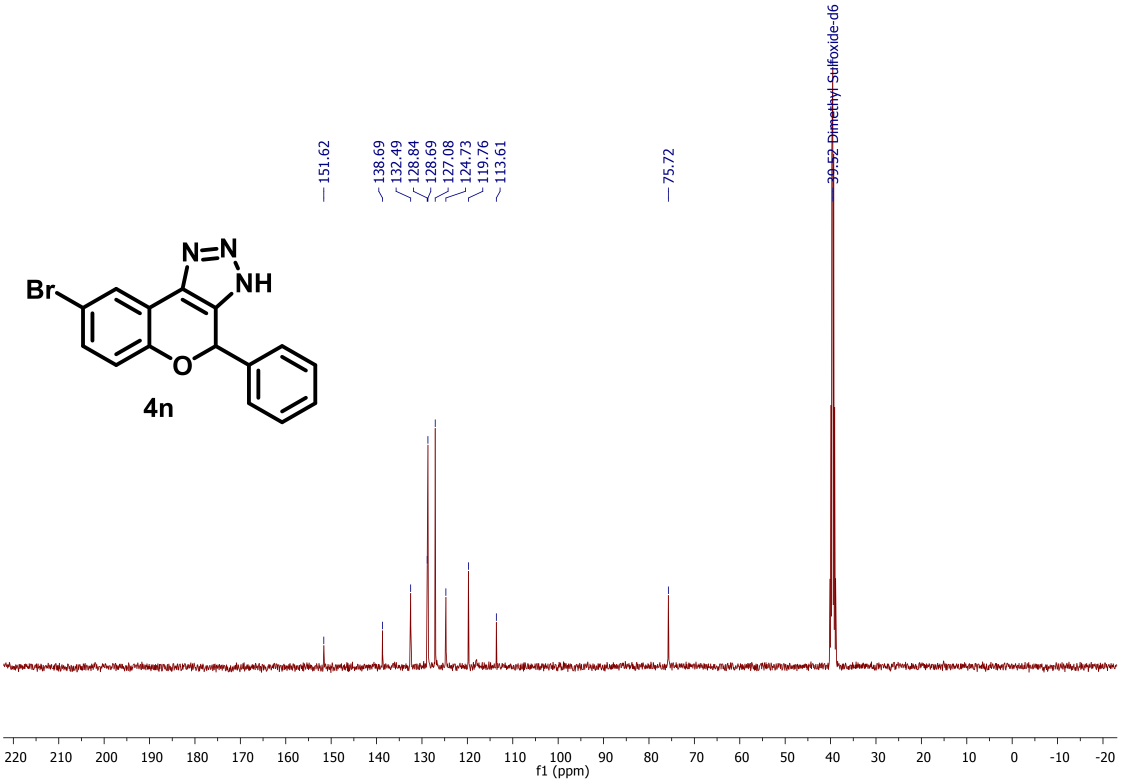

Figure 18. ^{13}C NMR spectra (100 MHz, DMSO-d_6) of compound **4n**.

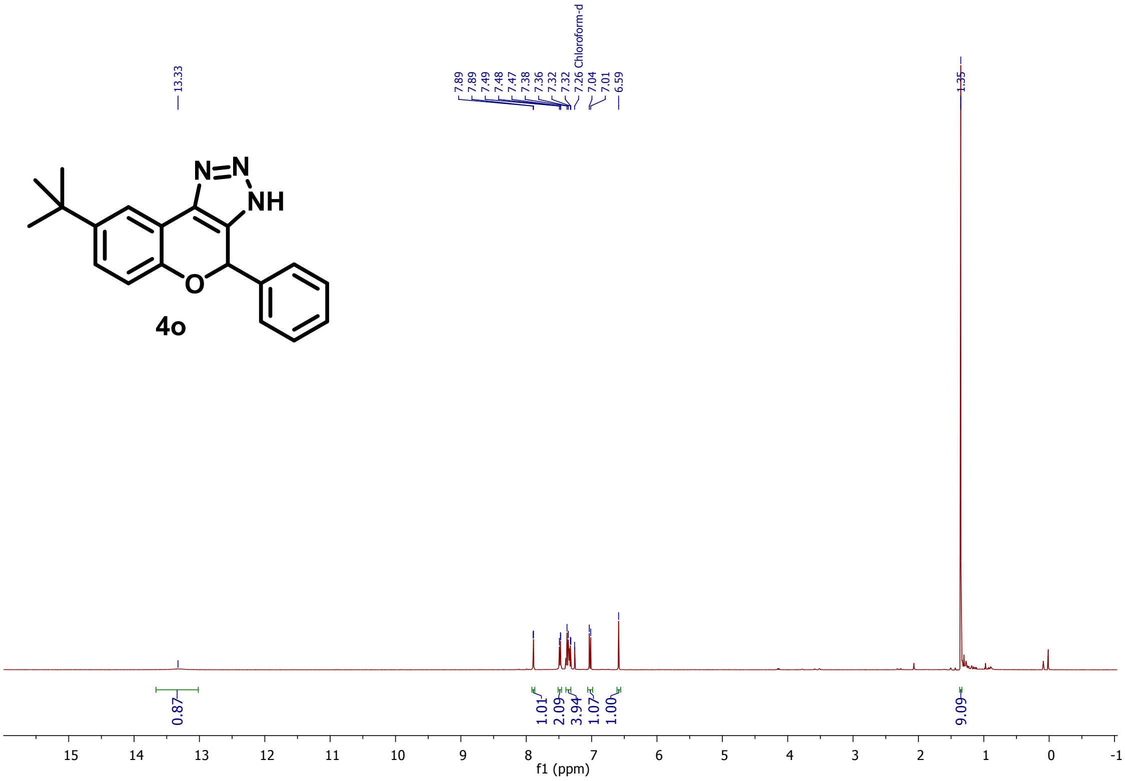

Figure 19 ^{1}H NMR spectra (400 MHz, $CDCl_3$) of compound **4o**.

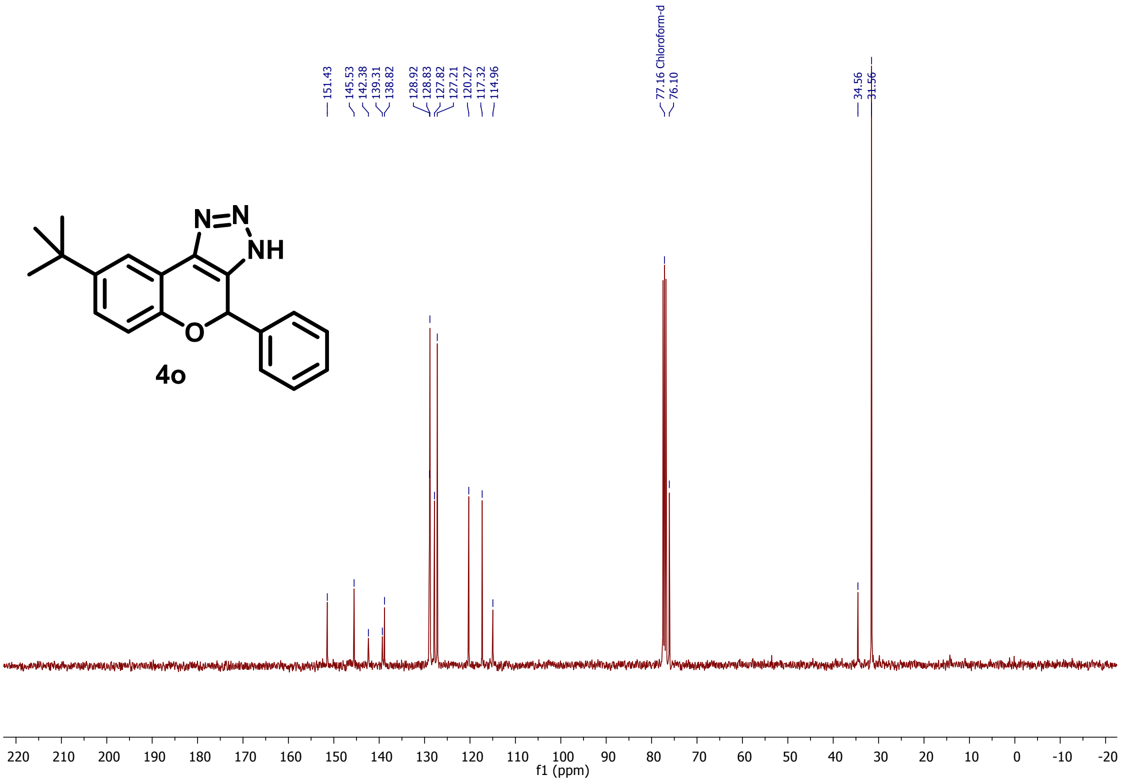

Figure 20. ^{13}C NMR spectra (100 MHz, $CDCl_3$) of compound **4o**.

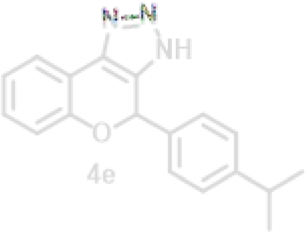

+MS, 0.80-1.09min #139-189

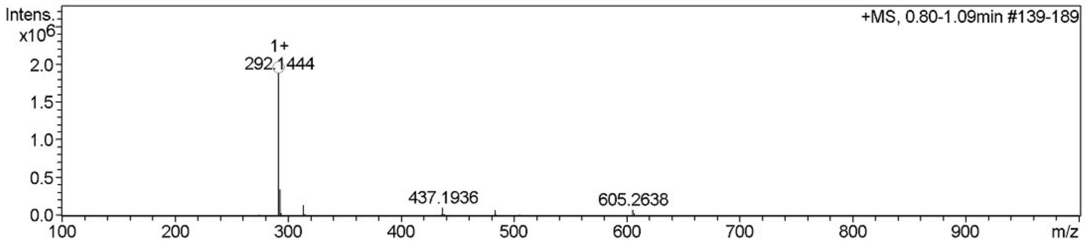

Meas. m/z	#	Ion Formula	Score	m/z	err [mDa]	err [ppm]	mSigma	rdb	e⁻ Conf	N-Rule	Adduct
292.144401	1	C18H18N3O	100.00	292.144439	0.0	0.1	11.7	11.5	even	ok	M+H

Figure 21. HRMS (ESI-TOF$^+$) of compound **4e**.

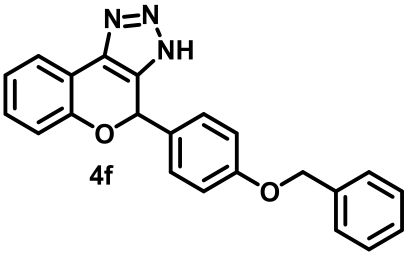

+MS, 0.78-1.04min #135-181

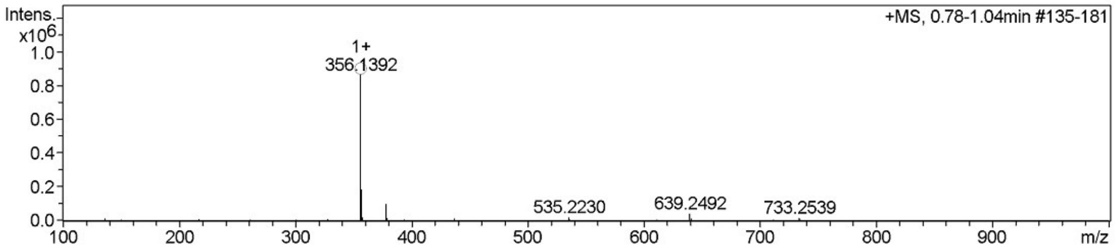

Meas. m/z	#	Ion Formula	Score	m/z	err [mDa]	err [ppm]	mSigma	rdb	e⁻ Conf	N-Rule	Adduct
356.139227	1	C22H18N3O2	100.00	356.139353	0.1	0.4	21.6	15.5	even	ok	M+H

Figure 22. HRMS (ESI-TOF$^+$) of compound **4f**.

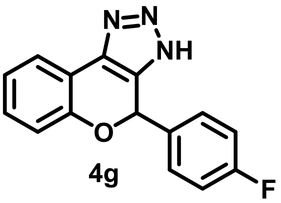

+MS, 0.80-0.98min #139-171

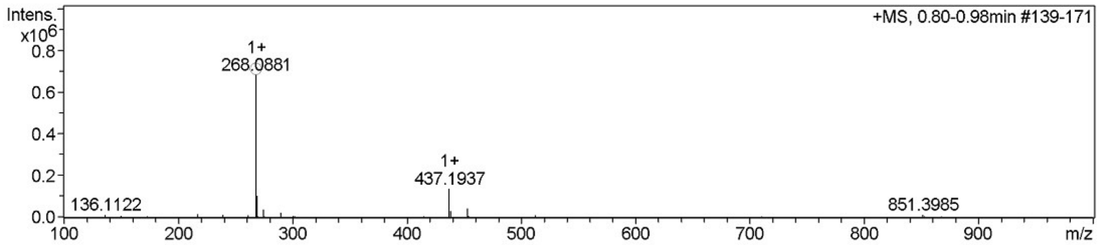

Meas. m/z	#	Ion Formula	Score	m/z	err [mDa]	err [ppm]	mSigma	rdb	e⁻ Conf	N-Rule	Adduct
268.088113	1	C15H11FN3O	100.00	268.088067	-0.0	-0.2	12.4	11.5	even	ok	M+H

Figure 23. HRMS (ESI-TOF$^+$) of compound **4g**.

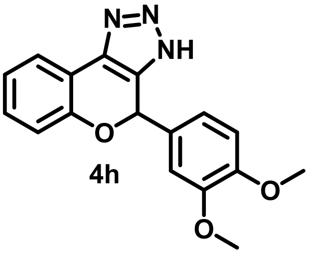

+MS, 0.70-0.91min #120-157

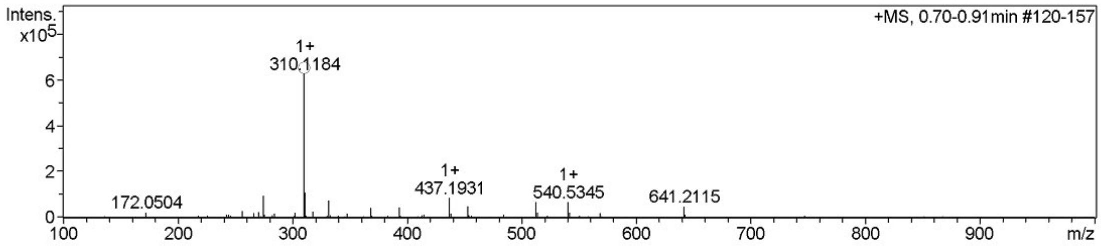

Meas. m/z	#	Ion Formula	Score	m/z	err [mDa]	err [ppm]	mSigma	rdb	e⁻ Conf	N-Rule	Adduct
310.118373	1	C17H16N3O3	100.00	310.118618	0.2	0.8	13.9	11.5	even	ok	M+H

Figure 24. HRMS (ESI-TOF$^+$) of compound **4h**.

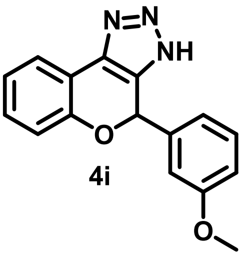

4i

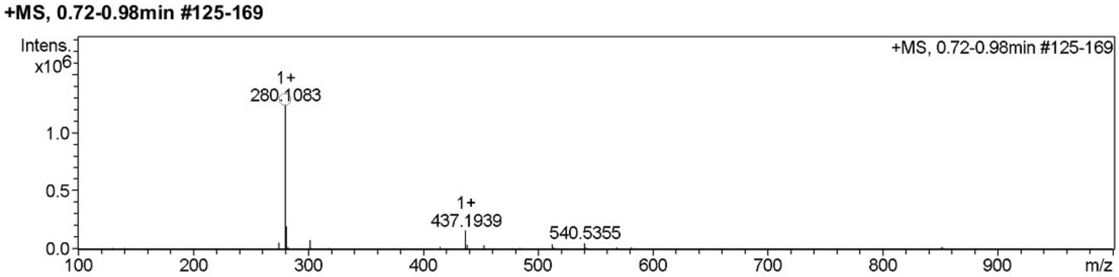

Meas. m/z	#	Ion Formula	Score	m/z	err [mDa]	err [ppm]	mSigma	rdb	e⁻ Conf	N-Rule	Adduct
280.108310	1	C16H14N3O2	100.00	280.108053	-0.3	-0.9	14.2	11.5	even	ok	M+H

Figure 25. HRMS (ESI-TOF^{+}) of compound **4i.**

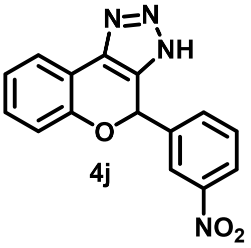

4j

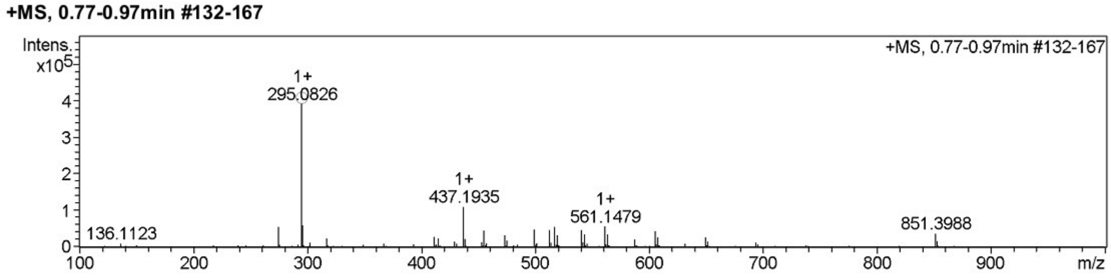

Meas. m/z	#	Ion Formula	Score	m/z	err [mDa]	err [ppm]	mSigma	rdb	e⁻ Conf	N-Rule	Adduct
295.082575	1	C15H11N4O3	100.00	295.082567	-0.0	-0.0	17.2	12.5	even	ok	M+H

Figure 26. HRMS (ESI-TOF^{+}) of compound **4j.**

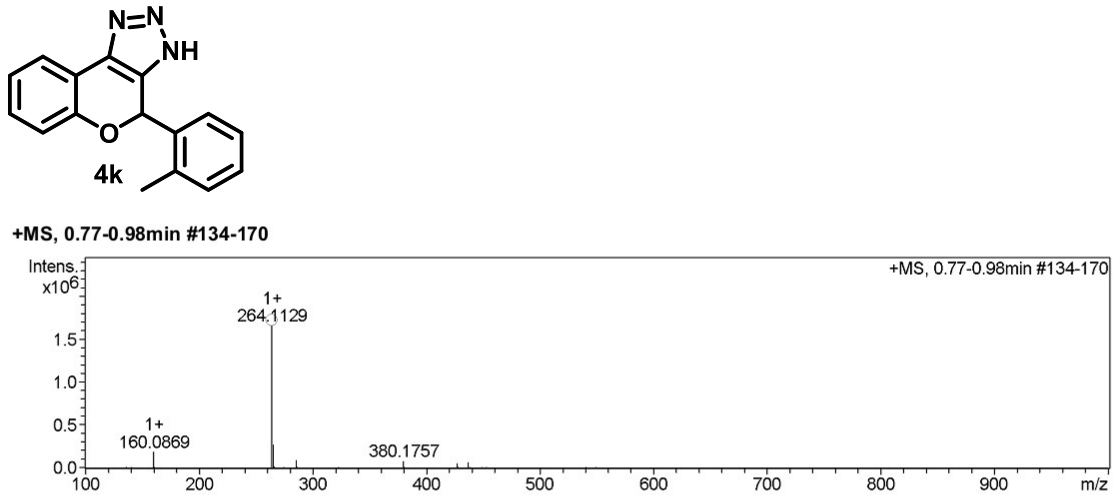

Meas. m/z	#	Ion Formula	Score	m/z	err [mDa]	err [ppm]	mSigma	rdb	e⁻ Conf	N-Rule	Adduct
264.112934	1	C16H14N3O	100.00	264.113139	0.2	0.8	10.4	11.5	even	ok	M+H

Figure 27. HRMS (ESI-TOF$^+$) of compound **4k.**

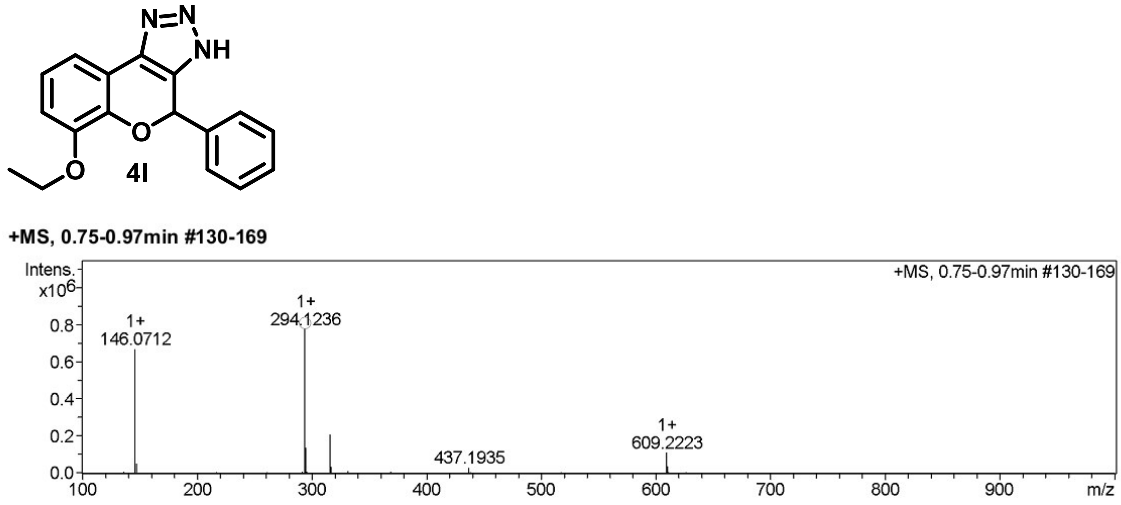

Meas. m/z	#	Ion Formula	Score	m/z	err [mDa]	err [ppm]	mSigma	rdb	e⁻ Conf	N-Rule	Adduct
294.123579	1	C17H16N3O2	100.00	294.123703	0.1	0.4	11.6	11.5	even	ok	M+H

Figure 28. HRMS (ESI-TOF$^+$) of compound **4l.**

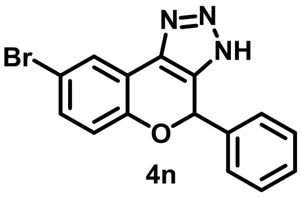

4n

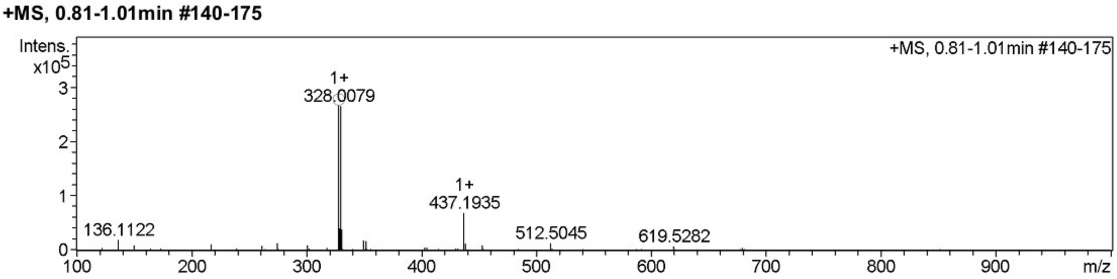

Meas. m/z	#	Ion Formula	Score	m/z	err [mDa]	err [ppm]	mSigma	rdb	e⁻ Conf	N-Rule	Adduct
328.007910	1	C15H11BrN3O	100.00	328.008001	0.1	0.3	18.3	11.5	even	ok	M+H

Figure 29. HRMS (ESI-TOF$^+$) of compound **4n.**

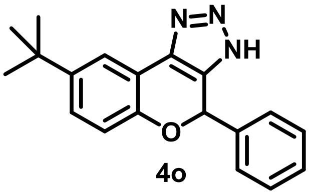

4o

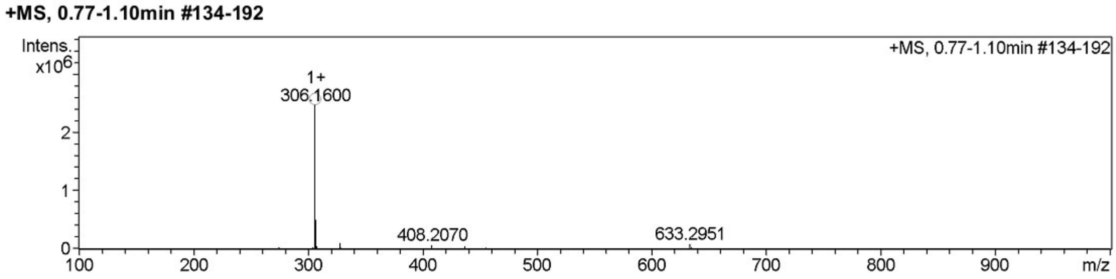

Meas. m/z	#	Ion Formula	Score	m/z	err [mDa]	err [ppm]	mSigma	rdb	e⁻ Conf	N-Rule	Adduct
306.159980	1	C19H20N3O	100.00	306.160089	0.1	0.4	8.3	11.5	even	ok	M+H

Figure 30. HRMS (ESI-TOF$^+$) of compound **4o.**

1. Furniss, B. S.; Hannaford, A. J.; Smith, P. W. G.; Tatchell, A. R.; *Vogel's "Pratical organic chemistry"*, 5a Ed., New York: Longman scientific & technical, **1989**, 1035-1036.

2. Chen, H.; Han, X.; Qin, N.; Wei, L.; Yang, Y.; Rao, L.; Chi, B.; Feng, L.; Ren, Y.; Wan, J.; *Bioorg. Med. Chem.*, **2016**, *24*, 1225–1230.

3. Rokade, B. V.; Prabhu, K. R.; *Org. Biomol. Chem.*, **2013**, *11*, 6713-6716.

4. Quan, X- J.; Ren, Z- H.; Wang, Y- Y.; Guan, Z- H.; *Org. Lett.*, **2014**, *16*, 5728− 5731.

5. Habib, P. M.; Raju, B. R.; Kavala, V.; Kuo, C.- W.; Yao, C. F.; Tetrahedron, **2009**, 65, 5799-5804.

6. (a) Trost, B. M.; *Sience*, **1991**, *254*, 1471-1477; (b) Trost, B. M.; *Angew. Chem. Int. Ed.*, **1995**, *34*, 259-281.

7. (a) Sheldon, R. A.; *Chem. Ind. (London)*, **1992**, 903-906; (b) Sheldon, R. A.; *Green. Chem.*, **2007**, *9*, 1273-1283.
